# Tongue Coating and the Salivary Microbial Communities Vary in Children with Halitosis

**DOI:** 10.1038/srep24481

**Published:** 2016-04-15

**Authors:** Wen Ren, Zhe Xun, Zicheng Wang, Qun Zhang, Xuenan Liu, Hui Zheng, Qian Zhang, Yifei Zhang, Linshuang Zhang, Chunyan Wu, Shuguo Zheng, Nan Qin, S. Dusko Ehrlich, Yihong Li, Xuesong He, Tao Xu, Ting Chen, Feng Chen

**Affiliations:** 1 Department of Preventive Dentistry, Peking University School and Hospital of Stomatology, Beijing, China; 2 Bioinformatics Division, TNLIST and Department of Automation, Tsinghua University, Beijing, China; 3 Central Laboratory, Peking University School and Hospital of Stomatology, Beijing, China; 4 Realbio Genomics Institute, Shanghai, China; 5 State Key Laboratory for Diagnosis and Treatment of Infectious Disease, The First Affiliated Hospital, College of Medicine, Zhejiang University, Hangzhou, China; 6 Metagenopolis, Institut National de la Recherche Agronomique, Jouy en Josas, France; 7 King’s College London, Centre for Host-Microbiome Interactions, Dental Institute Central Office, Guy’s Hospital, London Bridge, London, UK; 8 Department of Basic Science and Craniofacial Biology, New York University College of Dentistry, New York, USA; 9 School of Dentistry, University of California Los Angeles, Los Angeles, USA; 10 Programs in Computational Biology and Bioinformatics, University of Southern California, Los Angeles, USA

## Abstract

Halitosis is a common symptom mainly caused by microbial activities in the oral cavity. Here, we used *16S rRNA* gene pyrosequencing and metagenomic sequencing to examine oral microbial compositions and their functional variations in children with halitosis. We found that the tongue coating of subjects with halitosis had greater bacterial richness than those of healthy subjects. The relative abundance and prevalence of *Leptotrichia wadei* and *Peptostreptococcus stomatis* were higher in tongue coating samples from children with halitosis than those from children without halitosis; *Prevotella shahii* had higher relative abundance and prevalence in saliva samples from children with halitosis. We present the first comprehensive evaluation of the co-occurrence networks of saliva and tongue coating communities under healthy and halitosis conditions, and investigated patterns of significant differences between these communities. Moreover, we observed that bacterial genes associated with responses to infectious diseases and terpenoid and polyketide metabolism were enriched in subjects with halitosis, but not in healthy subjects. Hydrogen sulphide (H_2_S)-related metabolic pathways suggested that there was higher microbial production and less usage of H_2_S in subjects with halitosis. Thus, the mechanism of halitosis was implied for the first time via metagenomic sequencing.

Halitosis (also called oral malodour) is a condition characterized by foul-smelling or offensive odours emanating from the mouth or breath. This condition causes substantial personal discomfort and social limitations^1^. Studies indicate that 15–35% of adults^2^,^3^,^4^ and 14.5–40.9% of children^5^,^6^,^7^ worldwide suffer from halitosis. Owing to its high prevalence and negative social impact, halitosis has been ranked as the third most common reason for dental visits, after dental caries and periodontal disease^8^. Halitosis is a multifactorial condition; the factors involved vary according to the source of the malodour. Approximately 90% of halitosis cases are associated with intraoral conditions^9^, such as tongue coating, gingival and periodontal diseases, deep carious lesions, peri-implant diseases, exposed necrotic pulps or retention of food debris^10^,^11^. Extraoral halitosis is usually associated with respiratory diseases, gastrointestinal disorders or metabolic disorders^8^.

It is well accepted that the main cause of oral malodour is microbial degradation of cysteine, methionine, tryptophan, arginine and lysine into odiferous volatile sulphur compounds (VSCs) in the oral cavity^1^. VSCs in the oral cavity primarily comprise hydrogen sulphide (H_2_S), methyl mercaptan (CH_3_SH) and dimethyl sulphide [(CH_3_)_2_S]^12^. Historically, microbiological studies of halitosis used culture-based methods to detect pathogens. Several anaerobic bacteria and spirochetes, including *Bacteroides* spp., *Centipeda periodontii*, *Eubacterium* spp., *Fusobacterium nucleatum*, *Fusobacterium periodonticum*, *Selenomonas artemidis*, *Treponema denticola*, *Peptostreptococcus anaerobius*, *Porphyromonas endodontalis*, *Porphyromonas gingivalis*, *Prevotella intermedia*, *Prevotella loescheii* and *Solobacterium moorei*, have been identified as active producers of VSCs^9^,^13^,^14^,^15^,^16^. However, these and similar studies have excluded numerous as-yet-uncultured taxa owing to cultivation limitations. Now, because of the increased capabilities of next-generation sequencing (NGS)^17^ technology, studies of microbes in halitosis have shifted towards evaluating microbial communities that include uncultured taxa. For instance, Yang *et al*. used *16S rRNA* gene pyrosequencing to identify links between the oral microbiome and halitosis^18^, thereby providing comprehensive insight into the micro-ecological changes associated with halitosis. Nevertheless, to the best of our knowledge, previous studies of halitosis using NGS methods have focused mainly on adults. Surprisingly, little is known regarding the microbial communities that are associated with halitosis in children who have no other confounders; specifically, little is known about the gene compositions, functional significance and genetic variabilities of these microbial communities.

In this study, we collected tongue coating and saliva samples from children with and without halitosis and analyzed the microbial communities in these samples and functional variations among these communities using *16S rRNA* gene pyrosequencing and metagenomics. We sampled from the dorsum of the tongue because it is known to retain large quantities of microorganisms, exfoliated cells and as a result of its large and papillary surface area, it is well regarded as a major source of VSCs. Saliva, as a representative of the overall oral microbial population, is also associated with halitosis; in consideration of its host-friendly sampling accessibility, it was also collected in this study. Because children rarely have periodontal disease or systemic diseases, they are good candidates for studying halitosis. This study aimed to increase our understanding of the impact of oral microbes on halitosis in children.

## Results

### Halitosis tongue coating samples with a higher microbial richness than healthy samples

We performed oral examinations on 170 children ranging in age from 4 to 5 years old. The 10 subjects with the highest VSC concentrations were selected as the halitosis group. Children with normal VSC concentrations were considered “healthy” subjects in this study; ten of these subjects were randomly chosen as the healthy group (see Methods). The demographics and characteristics of the subjects are shown in Table S1. Tongue coating and saliva samples were collected from these 20 subjects and analysed using 454 pyrosequencing. One subject was excluded from the study (both tongue coating and saliva samples) owing to poor sequencing reads from his tongue coating sample. Thus, 38 samples remained. After processing with Mothur, we obtained a total of 394,697 final sequence reads with an average of 10,387 ± 1,907 reads per sample (Table S2). The 38 samples included a total of 1,436 operational taxonomic units (OTUs) at a 97% similarity cut-off, with 66–266 OTUs per sample. The rarefaction curves approached asymptotes for most samples (Fig. S2). In total, 12 phyla, 75 genera and 140 species were detected; the predominant taxa were similar to the taxa reported in previous studies^19^,^20^ and are shown in Fig. S3.

**Figure 1:**
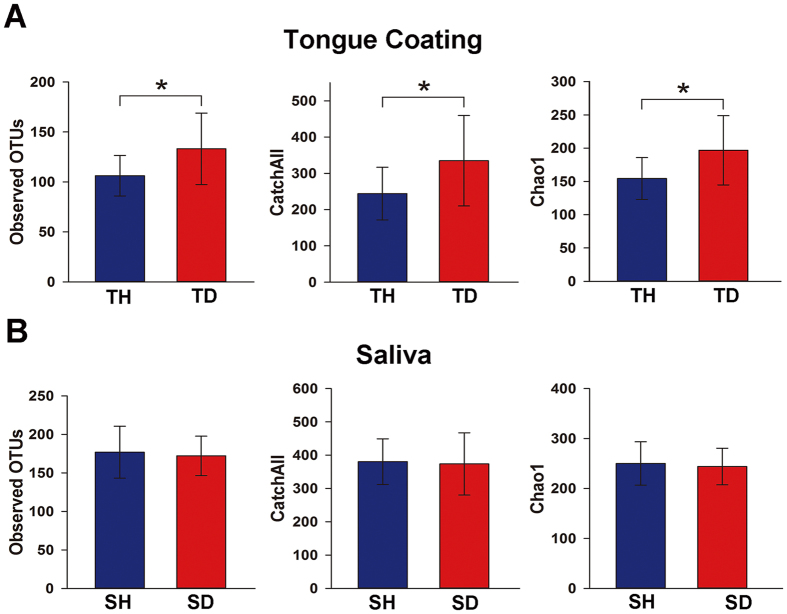
Comparisons of different α diversity indices in healthy and halitosis samples. A number of sequences (7,180) were randomly subsampled to obtain equal numbers of sequences from each dataset. (**A**) Observed OTUs, CatchAll (estimated number of OTUs) and Chao1, representing community richness, were calculated for the healthy tongue coating samples (TH) and the halitosis tongue coating samples (TD). (**B**) Observed OTUs, CatchAll and Chao1 comparisons between the healthy saliva (SH) and halitosis saliva samples (SD). *P < 0.05.

The α diversity indices (observed OTUs, CatchAll, and Chao1) were calculated from 7,180 reads (Table S3). They were significantly higher in the halitosis tongue coating samples than in the healthy tongue coating samples (P < 0.05; Fig. 1A), but they showed no significant difference between the healthy and halitosis saliva samples (P > 0.05; Fig. 1B). Phylogenetic community analysis based on the weighted UniFrac distance metric was used to examine the overall bacterial community composition of each group. The weighted UniFrac distance showed no difference between healthy and halitosis samples (tongue coating or saliva) (P > 0.05; Fig. S4), which is consistent with a previous study^18^.

### OTUs with different relative abundances and prevalences between healthy and halitosis samples

**Figure 2:**
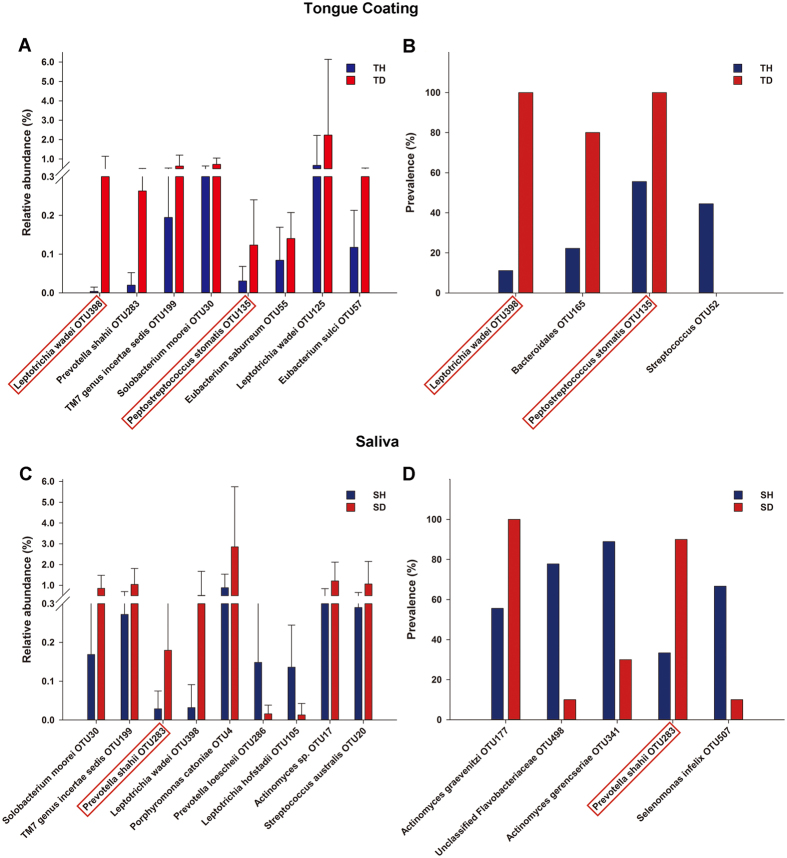
OTUs with different relative abundances and prevalences between the healthy and halitosis samples. (**A**) The graph depicts OTUs with different relative abundances and is based on Wilcoxon signed-rank test results from comparisons between healthy (TH) and halitosis tongue coating samples (TD). The bars represent the mean relative abundance (±SD). (**B**) OTUs with different prevalences were calculated based on Fisher’s exact test. Similar comparisons between the healthy (SH) and halitosis saliva samples (SD) are shown in (**C**) and (**D**). The OTUs framed by red boxes had different relative abundances and prevalences between the healthy and halitosis samples.

Next, we identified taxa that were responsible for the overall community differences between the healthy and halitosis samples. The relative abundances of 8 OTUs were significantly higher in the halitosis tongue coating samples (Fig. 2A). Notably, *Leptotrichia wadei* (OTU398) and *Peptostreptococcus stomatis* (OTU135) were detected in all of the halitosis tongue coating samples and were significantly more prevalent than in the healthy samples (Fig. 2B). The relative abundances of 9 OTUs differed significantly between the healthy and halitosis saliva samples; four of these OTUs (*L. wadei* (OTU398), *Prevotella shahii* (OTU283), *TM7 genus incertae sedis* (OTU199) and *Solobacterium moorei* (OTU30)) were also more abundant in the halitosis tongue coating samples (Fig. 2A,C). Only *P. shahii* (OTU283) was more prevalent in the halitosis saliva samples (Fig. 2D).

Principal component analysis (PCA) was implemented to assess discrepancies based on OTUs with different relative abundances (Fig. S5). Based on the PCA plot, we found that there was a tendency to form two clusters, a tight healthy cluster (Fig. S5, blue squares) and a disperse halitosis cluster (Fig. S5, red triangles), in both the tongue coating and saliva samples. Interestingly, two healthy samples (Fig. S5, blue squares marked with 4 and 5) fell within the halitosis cluster, indicating that these individuals may be at risk of halitosis.

### Distinct patterns in the co-occurrence networks of saliva and tongue coating communities under healthy and halitosis conditions

**Figure 3:**
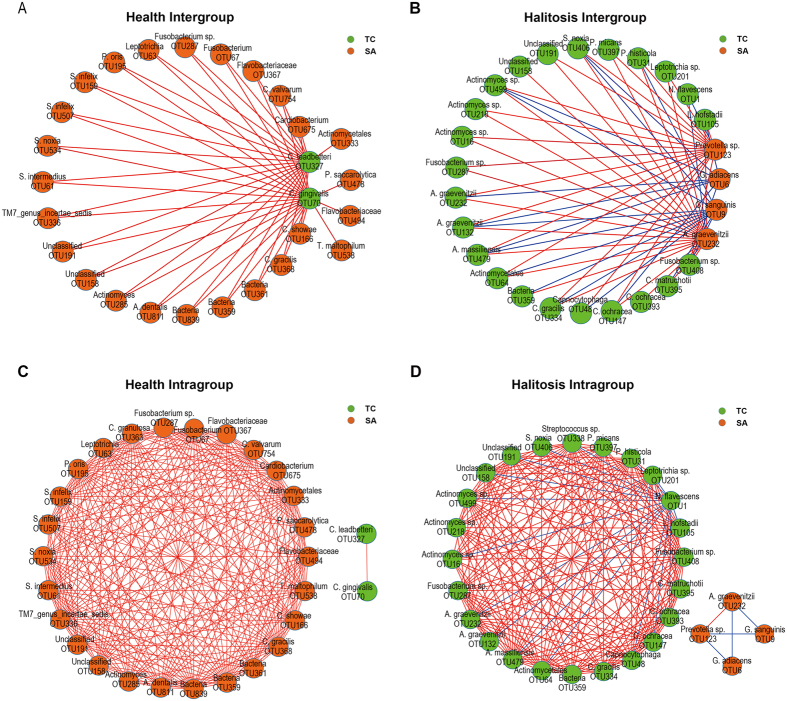
Co-occurrence networks between tongue coating and saliva samples under healthy and halitosis conditions. A co-occurrence network was constructed using the relative abundances of all OTUs with Pearson’s correlation coefficients |r| > 0.4 and Permutation test P < 0.01. OTUs with the most linkages (>30) were selected as the hub species. Each node represents an OTU. Lines between nodes show positive correlations (red) or negative correlations (blue). **(A**,**B)** Inter-group correlations (namely, correlations of OTUs between tongue coating and saliva) in healthy and halitosis groups. **(C**,**D)** Intra-group correlations (namely, correlations of OTUs within tongue coating samples and of OTUs within saliva samples) in healthy and halitosis groups. TC: tongue coating nodes, SA: saliva nodes.

By calculating and then visualizing the Pearson’s correlation coefficients for all of the OTUs in the healthy tongue coating and saliva samples, we generated a co-occurrence network of 924 OTUs with 3,907 edges. Nodes with more than 30 linkages (edges) were selected as hub nodes (Fig. 3). These correlations are divided into inter-group correlations (namely, correlations of OTUs between tongue coating and saliva), and intra-group correlations (namely, correlations of OTUs within tongue coating samples and of OTUs within saliva samples). Twenty-five saliva nodes together with only 2 tongue coating nodes were found in the healthy inter-group module; all of the correlations were positive (Fig. 3A). The two tongue coating nodes were both from the *Capnocytophaga* genus. Using the same method, twenty-three tongue coating nodes and 4 saliva nodes were found with 39 positive and 21 negative correlations in the halitosis inter-group module (Fig. 3B). Most of the halitosis hub tongue coating nodes were from the genera *Actinomyces*, *Capnocytophaga*, *Leptotrichia*, *Fusobacterium* and *Prevotella*, which have been previously associated with halitosis^14^,^15^,^18^,^21^,^22^,^23^. The characteristics of the hub nodes are shown in Table S4. The same patterns could be found in intra-group modules as well (Fig. 3C,D). These results suggested distinct patterns in the correlations of saliva and tongue coating communities under healthy and halitosis conditions.

### Variations in functional gene abundances of the H_2_S metabolic process between halitosis and healthy microbiomes

**Figure 4:**
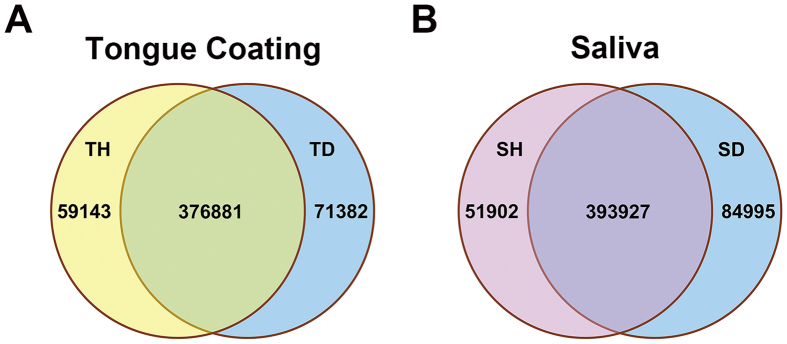
Comparisons of microbial gene sets in different samples. (**A**) Venn diagram of gene sets from a healthy tongue coating sample (TH) and a halitosis tongue coating sample (TD). The number of genes in each group and the overlapping area are shown. (**B**) Venn diagram of gene sets from a healthy saliva sample (SH) and a halitosis saliva sample (SD).

We generated 30.3 Gb of quality-filtered paired-end (PE) reads from four pooled metagenomic samples. Mapping of the reads to the human genome showed a low human DNA presence (<10%) in the tongue coating samples and a relatively high (>50%) human DNA presence in the saliva samples (Table S5). The reads were assembled into scaffolds, and 585,327 non-redundant genes were predicted within the assembled scaffolds (Table S6). By comparing the genes between the halitosis and healthy samples (Fig. 4) and between the tongue coating and saliva samples (Fig. S7), we found that most of the genes were shared by different samples. This gene set provided us with the ability to search for functions associated with halitosis.

**Figure 5:**
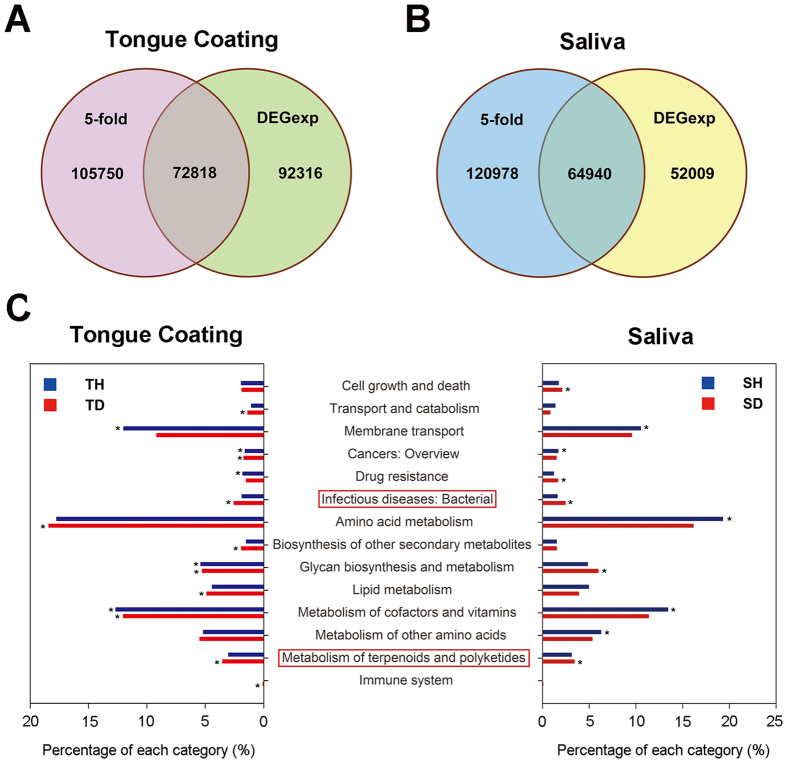
Comparisons of differential genes in tongue coating and saliva samples and enrichment of KO groups in tongue coating and saliva samples. (**A**) Genes with relative abundance differences of >5-fold and P-values < 0.001 in the DEGexp results were defined as differential genes in the tongue coating samples and are shown in the overlapping areas. (**B**) Differential genes in the saliva samples are shown. (**C**) KO enrichment groups in tongue coating and saliva samples are shown as percentages. Groups framed by a red box were enriched in both halitosis samples (tongue coating and saliva) but not in the healthy samples. *P < 0.05.

We searched for genes with differences in relative abundance between halitosis and healthy samples of >5-fold and with P-values for these differences of <0.001 in the DEGexp results^24^. There were 72,818 such genes in the tongue coating samples (38,428 (52.77%) were more abundant in the halitosis sample and 34,390 (47.23%) were more abundant in the healthy sample) (Fig. 5A) and 64,940 genes in the saliva samples (41,703 (64.22%) were more abundant in the halitosis sample and 23,237 (35.78%) were more abundant in the healthy sample) (Fig. 5B). The genes were then functionally annotated using the KEGG and eggNOG databases. Functional categories and genes in KEGG/eggNOG were counted (Figs S8 and S9), and KEGG ontology (KO) enrichment was analysed (Table S7). Interestingly, the groups “infectious diseases: bacterial” and “metabolism of terpenoids and polyketides” were enriched in the halitosis samples (both tongue coating and saliva samples), but not in the healthy samples (Fig. 5C). We suggest that microbial genes involved in infectious diseases might be relevant to halitosis. Terpenoids and polyketides are secondary metabolites produced by bacteria; they are structurally complex compounds involved in cell defence and communication. The involvement of these metabolites in halitosis was not previously suspected and warrants further investigation.

**Figure 6:**
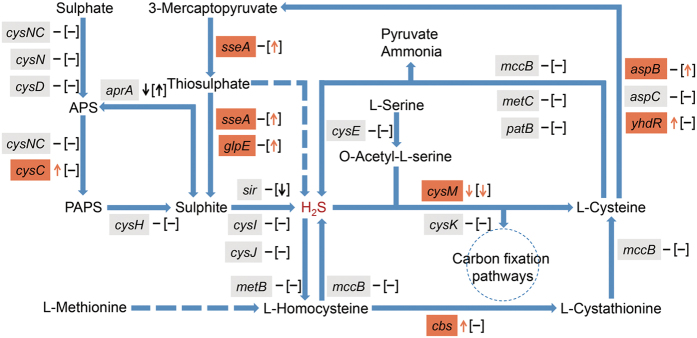
Functional variations in the H2S metabolic process between halitosis and healthy microbiomes. Genes with relative abundance ratios of >2 were defined as differentially abundant genes and are indicated by arrows. The upward arrows represent genes in the halitosis samples that were more abundant than in the healthy samples, whereas the downward arrows represent genes that were less abundant in the halitosis samples. Genes with relative abundance ratios of <2 are shown as “−”. Variations in tongue coating samples are labelled outside the “[]”, whereas variations in saliva samples are labelled inside the “[]”.

Additionally, the genes we assembled were mapped to the sulphur, cysteine and methionine metabolic pathways to explore hydrogen sulphide-related functional variations between halitosis and healthy microbiomes. We defined genes with relative abundance differences of >2-fold as differentially abundant genes. Previous studies have indicated that bacteria produce H_2_S in two ways: reduction of sulphate and desulphydration of cysteine and methionine^25^. Sulphate reduction is mediated by the enzymes encoded by the *cysNC*, *cysN*, *cysD*, *cysC*, *cysH*, *cysJ*, *cysI*, *aprA* and *sir* genes. Of these, *cysC* was more abundant in the halitosis tongue coating samples, whereas *sir* was less abundant in the halitosis saliva samples; no variations in the other genes were detected (Fig. 6, Table S8). The desulphydration process includes (1) reverse transsulphuration pathway activation via cystathionine β-synthase (encoded by *cbs*), cystathionine γ-lyase and homocysteine desulphydrase (encoded by *mccB*), which converts homocysteine (an essential intermediate in methionine oxidation and cysteine synthesis) into cystathionine, cysteine, pyruvate, ammonia, and H_2_S; and (2) cysteine transamination by aminotransferases (encoded by *aspB*, *aspC*, and *yhdR*) to yield 3-mercaptopyruvate, which is further metabolized by 3-mercaptopyruvate sulphurtransferase (encoded by *sseA*) and thiosulphate sulphurtransferase (encoded by *sseA* and *glpE*) to release sulphur^26^,^27^. Our study showed that *cbs*, *aspB*, *yhdR*, *sseA* and *glpE* were more abundant in the halitosis groups. Additionally, *cysM*, which encodes cysteine synthase B and catalyses the H_2_S-to-cysteine conversion in prokaryotes, was less abundant in the halitosis samples (Fig. 6, Table S8). This might result in higher H_2_S levels in the oral cavity compared with healthy subjects. To further clarify the bacterial contribution to the functional variations, we searched NCBI Bacterial Gene Database by using gene names (e.g., *cysC*) and their descriptions (e.g., adenylylsulfate kinase), and then computed the intersection part with the Kraken taxonomic profile. The candidate species are shown in the Table S9.

## Discussion

The human body harbours enormous quantities of microbes that reside in distinct habitats^28^. The oral cavity is one of the most important and complicated habitats in our body and supports diverse microbial communities which could be related to oral health or diseases. Previous evidences have shown that microbiome differences are often seen at the disease sites, for example, supragingival plaque for caries^20^ and subgingival plaque for periodontitis^29^. In studies on halitosis, tongue coating has been widely used, while saliva is also used when researchers focus on epidemiological characteristics in their studies^20^,^30^. In this research, our data on richness showed that differences were visible in tongue coating but not saliva (Fig. 1), which is consistent with the above view. Indeed, the presence of deep fissures in the tongue provides an environment with low oxygen levels, i.e., a relatively anaerobic niche, making halitosis-associated pathogens more likely to colonise.

Previous studies have found a number of halitosis-associated bacteria using different methods^13^,^15^,^18^,^21^,^31^. Some of our results agree with those of previous studies. For example, *S. moorei* is a Gram-positive bacterium that can convert cysteine into hydrogen sulphide^32^; it has been previously recognized as a pathogenic bacterium^15^,^16^,^31^. *S. moorei* (OTU30) was significantly more abundant in the halitosis samples compared to healthy samples (Fig. 2A,C), indicating an association with halitosis. Takeshita *et al*. found increased abundances of *Leptotrichia* in persons with oral malodour^33^, and Yang *et al*. reported that *L. wadei* was positively correlated with H_2_S concentrations^18^. Notably, we detected *L. wadei* (OTU398) in all of the halitosis tongue-coating samples but in only 10% of the healthy samples (Fig. 2B). This bacterium also showed a higher relative abundance in halitosis tongue coating samples (Fig. 2A). Thus, we inferred that *L. wadei* may be involved in halitosis. Persson *et al*. reported that periodontitis pathogens, such as *Po. gingivalis*, *Pr. intermedia*, *T. forsythensis*, *F. nucleatum* and *Tr. denticola*, have the capacity to produce VSCs *in vitro*^14^. These pathogens have also been recognized to have a positive correlation with oral malodour *in vivo*^22^,^34^,^35^. However, we found no differences in the abundances of any of these periodontitis pathogens between the healthy and halitosis groups. There are many possible reasons for this inconsistency. First, our study focused on preschool-aged children, whereas most other studies have focused on adults. Children rarely have periodontal diseases or systemic diseases and are all nonsmokers, thereby providing favourable conditions for a better understanding of halitosis-associated microbiota. Ling *et al*. reported that the diversity of the salivary microbiota in children was greater than in adults, indicating that oral cavity bacterial flora are dynamic and change during life^20^,^36^. Moreover, the prevalence of halitosis differs among races owing to diet, environment and host genetics^16^. Second, most previous studies have utilized culture-dependent methods or focused on only a small panel of anaerobic bacteria. However, approximately 600 bacterial species are present in the oral cavity, 35% of which cannot be cultivated at present^37^. In contrast to these conventional methods, we used *16S rRNA* gene pyrosequencing, a culture-independent method that can provide a broad view of the oral microbiota. Finally, in contrast to most previous studies, we used a gas chromatograph to test for halitosis. Although this method is time-consuming and complicated, it can distinguish among the 3 common sulphur compounds associated with halitosis and thus is suitable for a research environment^38^.

Instead of being caused by a single pathogen, halitosis may be caused by poly-microbial mutual effects^39^, in which microorganisms interact in a synergistic or opposing manner. We identified healthy and halitosis-associated modules of hub OTUs (Fig. 3). Twenty-five saliva nodes in inter-group correlations (twenty-six saliva nodes in intra-group correlations) and only 2 tongue coating nodes were identified as hub taxa in the healthy module, suggesting that saliva plays a more important role than tongue coating in maintaining microbial balance under healthy conditions (Fig. 3A,C). By contrast, this balance was disrupted in the halitosis module. Negative correlations were detected, which indicates that opposing relationships or colonization resistance may exist between species (Fig. 3B,D)^40^. The number of tongue coating hub nodes turned out to far exceed the number of saliva nodes. These changes between healthy and halitosis modules suggest that community interactions display different patterns in healthy and halitosis subjects. Furthermore, the predominant hub OTUs switched from saliva to tongue coating, indicating a role reversal.

**Figure 7:**
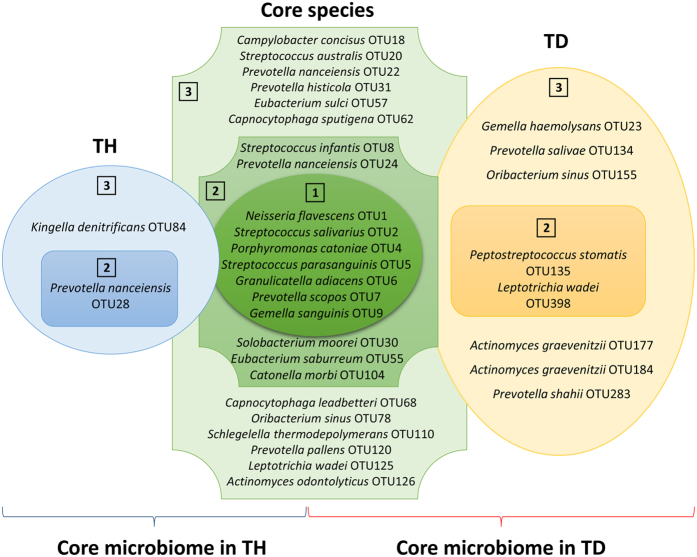
The core tongue coating microbiome and the health-associated and halitosis-associated tongue coating microbiomes. Core microbiomes were defined as OTUs with prevalences above 75% in both the healthy and halitosis groups (green). Health-associated microbiomes were defined as OTUs with prevalences above 75% in the healthy tongue coating samples, but less than 75% in the halitosis tongue coating samples (blue). Conversely, halitosis-associated microbiomes were defined as OTUs with prevalences above 75% in the halitosis tongue coating samples, but less than 75% in the healthy tongue coating samples (orange). Prevalence and relative abundance were used for further filtering. Circles labelled with “1” contain OTUs that were present in all 19 subjects with a mean relative abundance of ≥1% of the total sequences. Circles labelled with “2” contain OTUs that were present in all healthy (dark blue) or halitosis samples (dark orange), but with low abundances (<1%). The outer circles labelled with “3” contain OTUs with greater than 75% but less than 100% prevalence in the healthy (light blue) and halitosis samples (light orange) with a mean relative abundance of <1%. TH: healthy tongue coating sample, TD: tongue coating sample with halitosis.

Despite inter-individual variability, we detected several OTUs that were present in most of the tongue coating samples (Fig. 7). We defined these OTUs as the “core microbiome” of tongue coatings, thus representing the normal flora of this oral site (Fig. 7, green-coloured shape). Most of the species within the core microbiome of the tongue coating biofilm were Gram-negative anaerobic bacteria. These species are the most adaptable to the tongue coating environment. Similar definitions of core microbiomes in the healthy (TH) and halitosis (TD) tongue coatings were also determined (Fig. 7, blue and orange shapes). Within the TD core microbiome, *L. wadei* (OTU398), *P. stomatis* (OTU135) and *P. shahii* (OTU283) were candidate halitosis pathogens, as discussed above (Fig. 2A,B). Taxonomic analysis was also performed with the metagenomic sequencing data using a relatively new method called “Kraken”, which is an ultrafast and highly accurate program for assigning taxonomic labels^41^. The metagenomic taxa were then compared with the Human Oral Microbiome Database (HOMD)^37^,^42^ at the genus and species levels, as HOMD is considered a powerful oral microbiome reference and contains more than 600 prokaryote species. We identified 605 genera and 1,274 species, including more than 196 genera and 708 species in HOMD. The number of species we identified is consistent with Jenkinson’s view that the estimated species number might turn out to be higher (~1200) than that in the database^43^. Therefore, metagenomic sequencing helps identify a microbiota that is broader than the current database.

Of the three major VSCs involved with oral malodour, (CH_3_)_2_S is the main contributor to extra-oral halitosis^44^, whereas CH_3_SH is more pathogenic than H_2_S in halitosis and is associated with periodontal disease^10^,^12^. Therefore, we mainly focused on H_2_S in the present study. We attempted to identify candidate halitosis pathogens using *16S rRNA* gene pyrosequencing; however, the most differentially abundant OTUs lacked evidence of H_2_S production *in vitro* (Fig. 2). We thus inferred that the pathogenesis of halitosis might be due to cooperative poly-microbial interactions rather than the effect of a single pathogen. Belda-Ferre *et al*. first used metagenomic approach in oral samples^45^, which could provide a convenient method for comprehensively studying poly-microbial interactions with functional information. By analysing the H_2_S metabolic process in the metagenomic data, we revealed functional differences between the halitosis and healthy microbiomes. As expected, there were significant increases in the abundances of the *cbs*, *aspB*, *yhdR*, *sseA* and *glpE* genes, which are involved in the reverse transsulphuration pathway and in the cysteine transamination pathway, which converts amino acids into H_2_S^26^,^27^. This result is consistent with previous studies showing that microbial degradation of methionine and cysteine in the oral cavity is the main cause of halitosis^8^,^46^. Moreover, the reduced abundance of *cysM* in our study might represent reduced conversion of H_2_S during cysteine metabolism in prokaryotes, resulting in lower usage of H_2_S. The higher production and lower use could contribute to excessive H_2_S concentrations and, hence, halitosis. In our study, few differences were found in most of the VSC pathway genes. Some of these genes might be present at equal frequencies but differentially expressed; metatranscriptomics are required to identify such differences.

In summary, this study explored microbial composition and function in preschool-aged children with and without halitosis. The study identified several OTUs that differed significantly in relative abundance and frequency of detection, thereby providing a list of candidate halitosis-associated pathogens. Moreover, this study offered the first comprehensive evaluation of co-occurrence networks of saliva and tongue coating communities under healthy and halitosis conditions and investigated significantly different patterns. Additionally, an H_2_S-related pathway was constructed to analyse the pathogenesis of halitosis. However, the aetiology of halitosis is more complex than expected, and further experiments are required to gain a better understanding of the condition.

## Methods

### Ethics statement

This project was approved by the Ethics Committee of the Peking University School and Hospital of Stomatology (PKUSSIRB-2012062). All the experiments were performed in accordance with relevant guidelines and regulations. All the subjects’ guardians provided written informed consent for participation.

### Enrolment criteria

We performed oral examinations on 170 children from YaYuncun No. 2 Kindergarten, Beijing, China (aged 4–5 years). OralChroma^TM^ (CHM-1, ABILIT Corporation, Japan) was used to measure concentrations of H_2_S, CH_3_SH, and (CH_3_)_2_S in the breath after calibration by the manufacturer. Each child was examined using OralChroma^TM^ 3 times, and the average values were used as criteria for diagnosis. The normal reference values of H_2_S, CH_3_SH and (CH_3_)_2_S were 1.50 ng 10 ml^−1^, 0.50 ng 10 ml^−1^, and 0.20 ng 10 ml^−1^, respectively^47^. A child was considered to have halitosis if any of these three compounds had an above-normal value. The 10 subjects with the highest VSC concentrations were selected as the halitosis group. Children with normal VSCs concentrations were considered “healthy” subjects; ten of these subjects were randomly chosen as the healthy group. None of these subjects presented with the common cold, respiratory diseases, digestive diseases or systemic diseases and none had taken antibiotics in the three months prior to the study. Decayed, missing, and filled teeth (dmft), tongue coating area (TCA), tongue coating thickness (TCT), gingival index (GI), debris index-simplified (DI-S), and saliva pH were also examined. All of the examinations and diagnoses were performed by one experienced dentist.

### Sample collection

Tongue coating and saliva samples were collected from the 20 subjects. To collect tongue coating samples, a sterile toothbrush was used to gently scrape the tongue dorsum from the vallate papilla area to the front tongue border. The toothbrush was then swirled in a 50-ml centrifuge tube containing 10 ml of TE (50 mM Tris-HCl and 1 mM EDTA; pH 7.6). A total of 1 ml of paraffin gum-stimulated saliva was collected in an empty 5-ml sterile Eppendorf tube. All samples were immediately frozen at −20°C and stored at −80°C prior to DNA extraction.

### *16S rRNA* gene pyrosequencing

#### DNA extraction, PCR amplification and 454 pyrosequencing

DNA was extracted using a TIANamp Bacteria DNA Kit (Tiangen Biotech, Beijing, China) following the manufacturer’s instructions. DNA purity was evaluated based on A260/A280 ratio using a NanoDrop 8000 Spectrophotometer (Thermo Fisher Scientific, USA). DNA integrity was verified by agarose gel electrophoresis. A negative control containing only buffer was included during DNA extraction and quantification. DNA samples were stored at −20°C prior to use.

Amplicon libraries of the *16S rRNA* gene V1-V3 hypervariable regions were generated using the universal primers 27F (5′-AGAGTTTGATCCTGGCTCAG-3′) and 534R (5′-TTACCGCGGCTGCTGGCAC-3′)^19^. PCR was performed as described in the manual for the GS FLX Amplicon DNA library preparation method (Roche, Mannheim, Germany). The cycling conditions were as follows: initial denaturation at 94 °C for 3 min; 30 cycles of denaturation at 94 °C for 30 s, annealing at 57 °C for 45 s, and extension at 72 °C for 1 min; and a final extension at 72 °C for 2 min. A DNA isolation negative control and a PCR control without template were included. The results of the PCR amplification were assessed by electrophoresis of a 1% agarose gel. The *16S rRNA* gene PCR amplicons were sequenced with a 454 GS FLX Titanium system (454 Life Sciences, Branford, CT, USA) at the BGI Institute (Shenzhen, China).

#### 454 sequence data processing

Sequence data were processed using Mothur^48^. The sequences were demultiplexed using a unique barcode assigned to each sample. The sequences were filtered according to quality scores using the sliding window approach, in which sequences are trimmed when the average quality score over a 50-nt sliding window drops below 30. A maximum of one barcode mismatch and two primer mismatches were allowed. Sequences >200 nt in length were retained. The sequences were further denoised using a single-linkage algorithm, and chimaeric sequences were removed using the UCHIME algorithm in Mothur. Unique sequences were taxonomically assigned with the RDP classifier^49^ using a bootstrapping algorithm. The bootstrapping threshold was 0.8; bootstrap values below 0.8 were assigned as “unclassified”. Unique tags were pre-clustered using a pseudo-single linkage algorithm to diminish errors caused by pyrosequencing. Sequences were clustered using the average neighbour algorithm for operational taxonomic unit (OTU) analysis with a 3% dissimilarity cut-off^50^. OTUs were assigned a taxonomy based on the consensus taxonomic assignment for the majority of sequences within that OTU. If a consensus taxonomy assignment was not possible at the species level, then the OTU was assigned at the lowest possible taxonomic level.

Rarefaction curves were generated with Mothur to test the current sequencing depth. Observed OTUs, CatchAll, and Chao1 were used to estimate the α diversity. The communities were compared based on phylogenetic distances using the weighted UniFrac metric to represent β diversity^51^. A Venn diagram was constructed to describe the core microbiome, healthy subject-associated species and halitosis-associated species. The relative abundances and prevalences of OTUs and different species were calculated.

#### Statistical analysis

The independent Student’s *t*-test and AMOVA were applied to evaluate α and β diversity. The Wilcoxon rank-sum test and Fisher’s exact test were used to examine relative abundance and prevalence, respectively. P < 0.05 indicated statistical significance. Principal component analysis (PCA) was performed based on OTUs with different relative abundances. Pearson’s correlation coefficients (PCC) were calculated between the OTUs in healthy tongue coating and saliva samples and between the OTUs in halitosis tongue coating and saliva samples^40^; the permutation test was then used to examine the significance of the PCC values (P < 0.01 was considered statistically significant). Co-occurrence networks were generated using Cytoscape (version 3.3.1). Student’s *t*-test, the Wilcoxon rank-sum test, Fisher’s exact test, Pearson’s correlation coefficients and the permutation test were performed using R (version 2.15.3).

### Metagenomics

#### DNA library construction and metagenomic sequencing

We first pre-evaluated human DNA from each sample using a qPCR method described in a previous report by Wang^52^. We found that human DNA contaminations of samples within the same group had similar proportions, indicating similar proportions of the microbial DNA in the samples of each group. Therefore, we pooled equimolar genomic DNA from each sample from each group to create new samples. In doing this, we created a total of four metagenomic samples. The quantity and quality of the metagenomic DNA were measured using a NanoDrop 2000 spectrophotometer (Thermo Fisher Scientific, USA) and by agarose gel electrophoresis (0.8%, 120 V), respectively. Next, DNA libraries were constructed according to the standard protocol provided by the manufacturer (Illumina, USA). Quality control of the libraries was performed on a StepOne Plus Real-Time PCR system (ABI, USA) prior to sequencing. The four libraries were sequenced in one lane using an Illumina HiSeq 2500 instrument (Illumina, USA) with 2 ×150 base pair (bp) paired-end (PE) sequencing. Library construction and sequencing were conducted at BerryGenomics (Beijing, China).

#### Quality control of reads and human contamination evaluation

We performed quality control of the sequencing data as follows: (1) reads containing more than 3% N bases were removed, (2) reads containing more than 50% bases with low quality (<3) were removed. Then, clean reads that mapped to the human genome (hg19) were filtered from each sample using Bowtie 2 with default parameters^53^.

#### Taxonomic analysis

All clean reads were aligned to the NCBI bacteria, archaebacteria and virus genome database using Kraken^41^ with default parameters. After achieving a taxonomy profiling table, we compared the taxa to the Human Oral Microbiome Database (HOMD)^37^,^42^ at the genus and species levels.

#### De novo assembly and gene prediction

All microbial PE reads were assembled by SOAPdenovo with default parameters^54^. The MetaGeneMark gene prediction tool was used with default parameters to predict genes in the assembled scaffolds^55^. The predicted open reading frames (ORFs) were compared against the NCBI non-redundant^56^ sequence database using BLAT with default parameters.

#### Functional analysis

We calculated the relative abundances of genes and then calculated the relative abundance ratios between the halitosis and healthy samples. DEGexp, a method in the DEGseq package^24^, was also used to select genes that differed between the halitosis and healthy samples, although this approach has mainly been used for RNA-seq data. DEGexp was used here because it could correct inexact relative abundance ratios caused by samples with very low abundance. Genes with relative abundance differences of >5-fold and with P-values < 0.001 in the DEGexp results were functionally annotated using the Kyoto Encyclopedia of Genes and Genomes (KEGG) bioinformatics database (8^th^ KEGG release, December 2014)^57^ and the eggNOG 4.0 database^58^. Functional categories and genes in KEGG/eggNOG were counted. Differential genes were subjected to KEGG Ontology (KO) enrichment analysis, and enriched P-values were calculated according to the hypergeometric test (P < 0.05 was considered statistically significant). Furthermore, genes that mapped to the sulphur metabolic pathway and to the cysteine and methionine metabolic pathway were analysed. Hydrogen sulphide-related genes were used to construct an H_2_S metabolic map. We defined genes with relative abundance ratios of >2 as differentially abundant genes in the H_2_S metabolic analysis. To determine taxonomic origins of these genes, we searched the NCBI Bacterial Gene Database by using gene names and gene descriptions, followed by computing the intersection with the Kraken taxonomic profile.

The sequencing data from this study have been submitted to Sequence Read Archive (http://www.ncbi.nlm.nih.gov/sra/) under accession SRX831098 and SRX831194 for *16S rRNA* gene pyrosequencing and metagenomic sequencing.

This manuscript was edited for English language usage, grammar, spelling and punctuation by native English-speaking editors at NPG Language Editing. For a certificate, please see: https://languageediting.nature.com/, key: B460-76A7-2AE1-22C2-A220.

## Additional Information

**How to cite this article**: Ren, W. *et al*. Tongue Coating and the Salivary Microbial Communities Vary in Children with Halitosis. *Sci. Rep*. **6**, 24481; doi: 10.1038/srep24481 (2016).

## Supplementary Material

Supplementary Information
