# Supplementary material for: Tongue Coating and the Salivary Microbial Communities Vary in Children with Halitosis
Source: Sci Rep. 2016 Apr 15;6:24481. doi: 10.1038/srep24481 (PMC4832241; doi:10.1038/srep24481)
Supplement: Supplementary Information [file srep24481-s1.pdf]

Tongue Coating and the Salivary Microbial Communities Vary in Children with Halitosis

Wen Ren[1,#], Zhe Xun[1,#], Zicheng Wang[2], Qun Zhang[1], Xuenan Liu[1], Hui Zheng[3], Qian Zhang[3], Yifei Zhang[3], Linshuang Zhang[4], Chunyan Wu[4], Shuguo Zheng[1], Nan Qin[5], S. Dusko Ehrlich[6,7], Yihong Li[8], Xuesong He[9], Tao Xu[1,*], Ting Chen[2,10,*], Feng Chen[3,*]

[#] These authors contributed equally to this work.

[1] Department of Preventive Dentistry, Peking University School and Hospital of Stomatology, Beijing, China, [2] Bioinformatics Division, TNLIST and Department of Automation, Tsinghua University, Beijing, China, [3] Central Laboratory, Peking University School and Hospital of Stomatology, Beijing, China, [4] Realbio Genomics Institute, Shanghai, China, [5] State Key Laboratory for Diagnosis and Treatment of Infectious Disease, The First Affiliated Hospital, College of Medicine, Zhejiang University, Hangzhou, China, [6] Metagenopolis, Institut National de la Recherche Agronomique, Jouy en Josas, France, [7] King's College London, Centre for Host-Microbiome Interactions, Dental Institute Central Office, Guy's Hospital, London Bridge, London, UK, [8] Department of Basic Science and Craniofacial Biology, New York University College of Dentistry, New York, USA, [9] School of Dentistry, University of California Los Angeles, Los Angeles, CA, USA, [10] Programs in Computational Biology and Bioinformatics, University of Southern California, Los Angeles, USA.

* Corresponding authors:

Feng Chen: 22# Zhongguancun Nandajie, Haidian District, Beijing 100081, China. Tel: +86-010-82195773. Email: moleculecf@gmail.com;

Ting Chen: FIT 1-107, Tsinghua University, Beijing, 100084, China. Tel: +86-010-62797101. Email: tingchen@mail.tsinghua.edu.cn;

Tao Xu: 22# Zhongguancun Nandajie, Haidian District, Beijing 100081, China. Tel: +86-010-82195269. Email: taoxu@bjmu.edu.cn.

Supplementary Figures

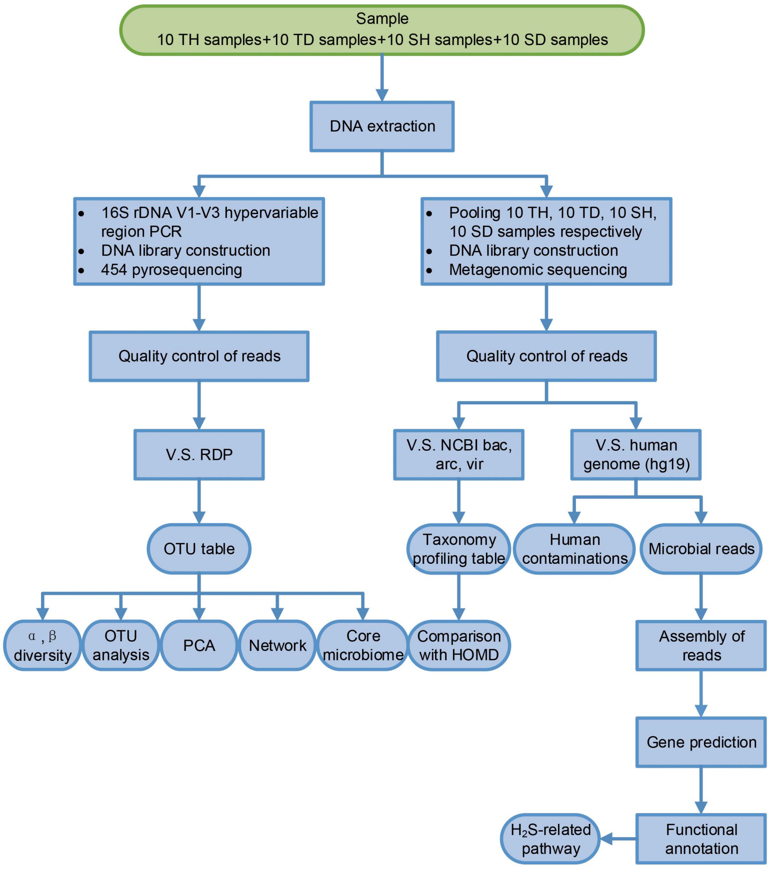

Figure S1. Experimental process. TH: healthy tongue coating sample, TD: tongue coating sample with halitosis, SH: healthy saliva sample, SD: saliva sample with halitosis.

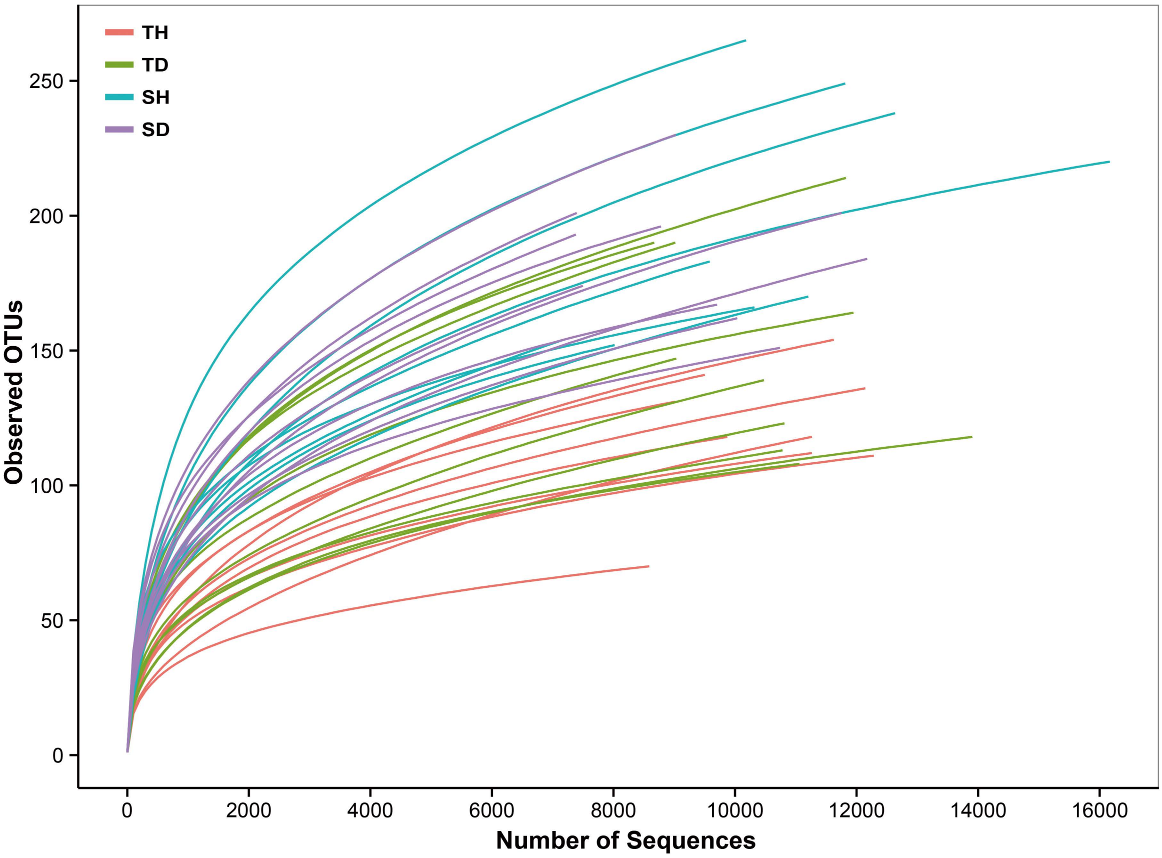

Figure S2. Rarefaction curves of 38 samples. Every 100 sequences were randomly sampled in each dataset. Most curves become flat in the end indicating that a reasonable number of tags were analyzed.

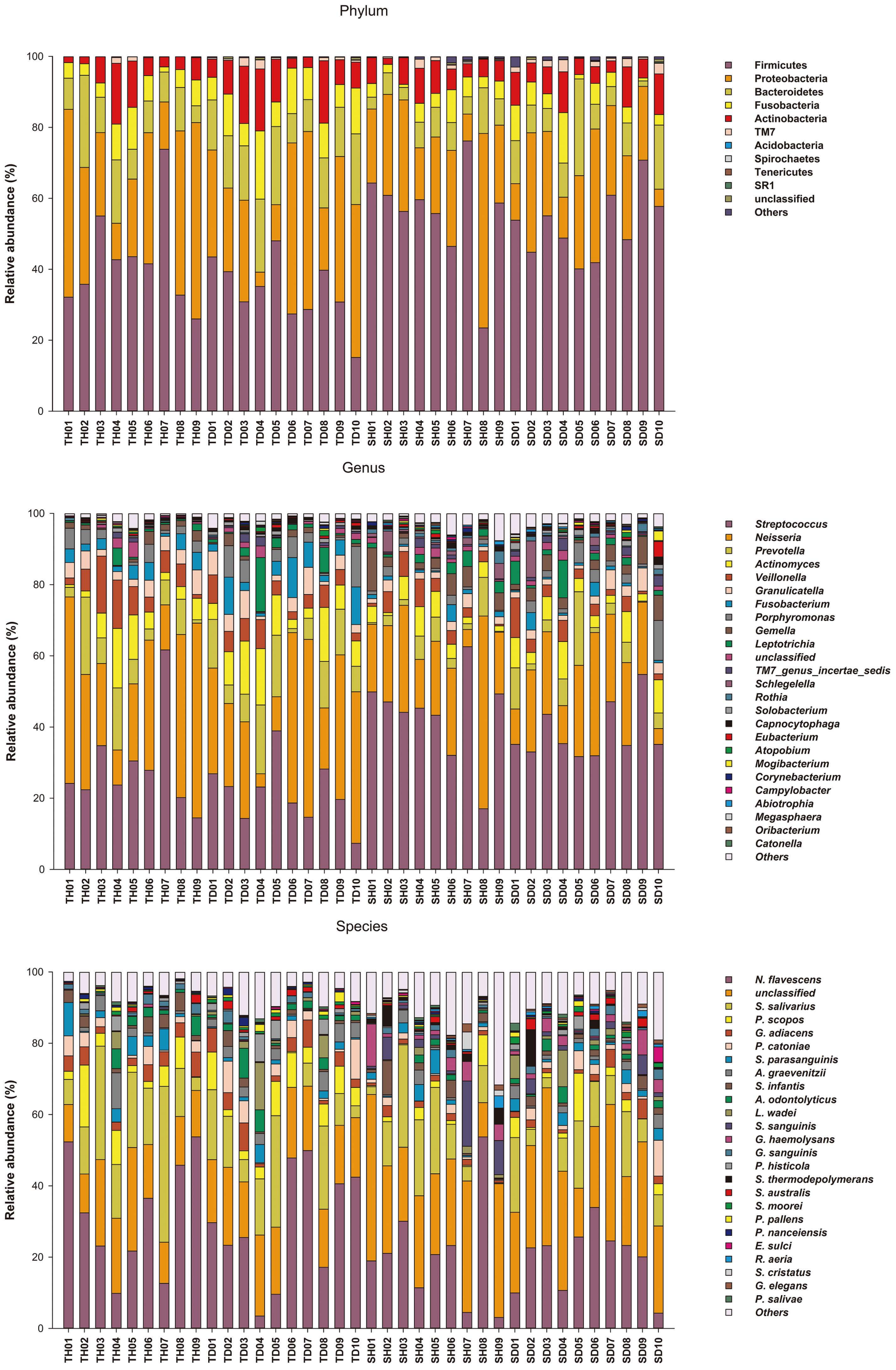

Figure S3. Community composition of each sample at phylum, genus and species level. All the phyla, the predominant 25 genera and species are shown. Label on x-axis represents sample ID.

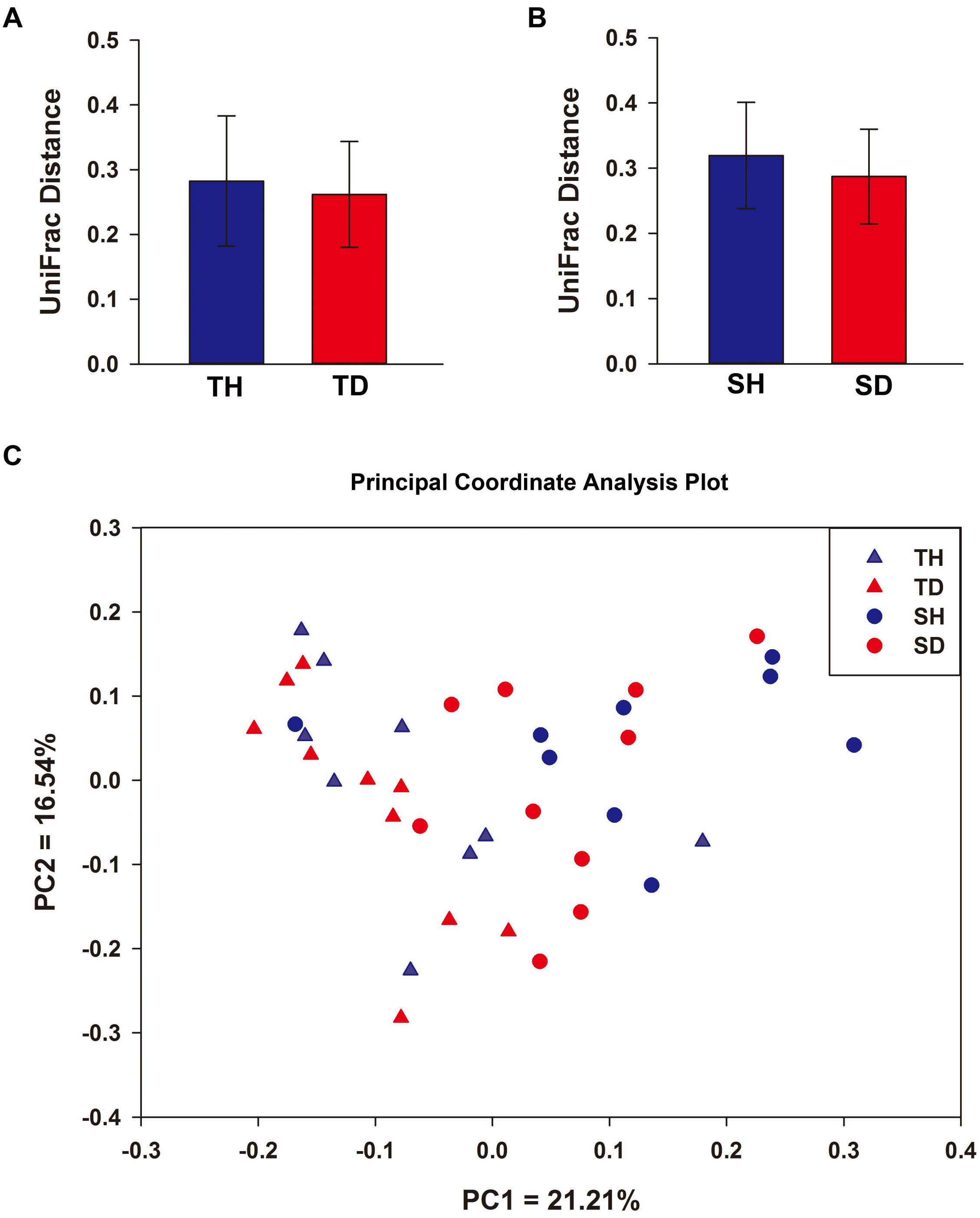

Figure S4. Comparisons of β diversity calculated using weighted UniFrac distance.

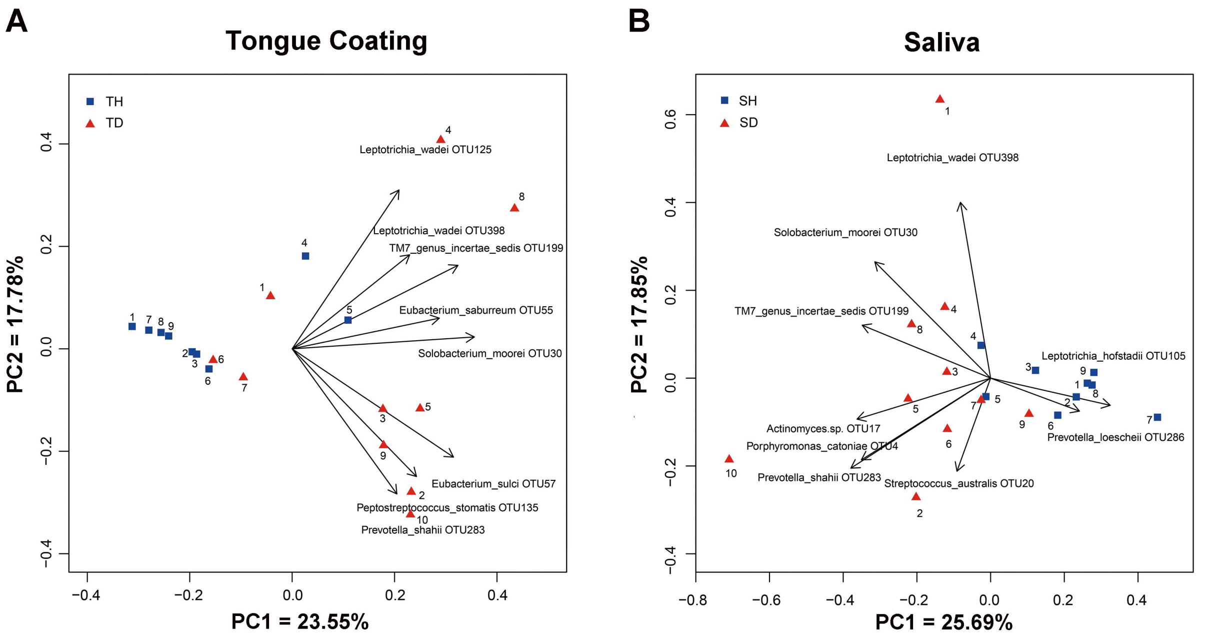

Figure S5. Results of principal component analysis (PCA). PCA plots were generated using OTUs with different relative abundances **(A)** between TH and TD, **(B)** between SH and SD.

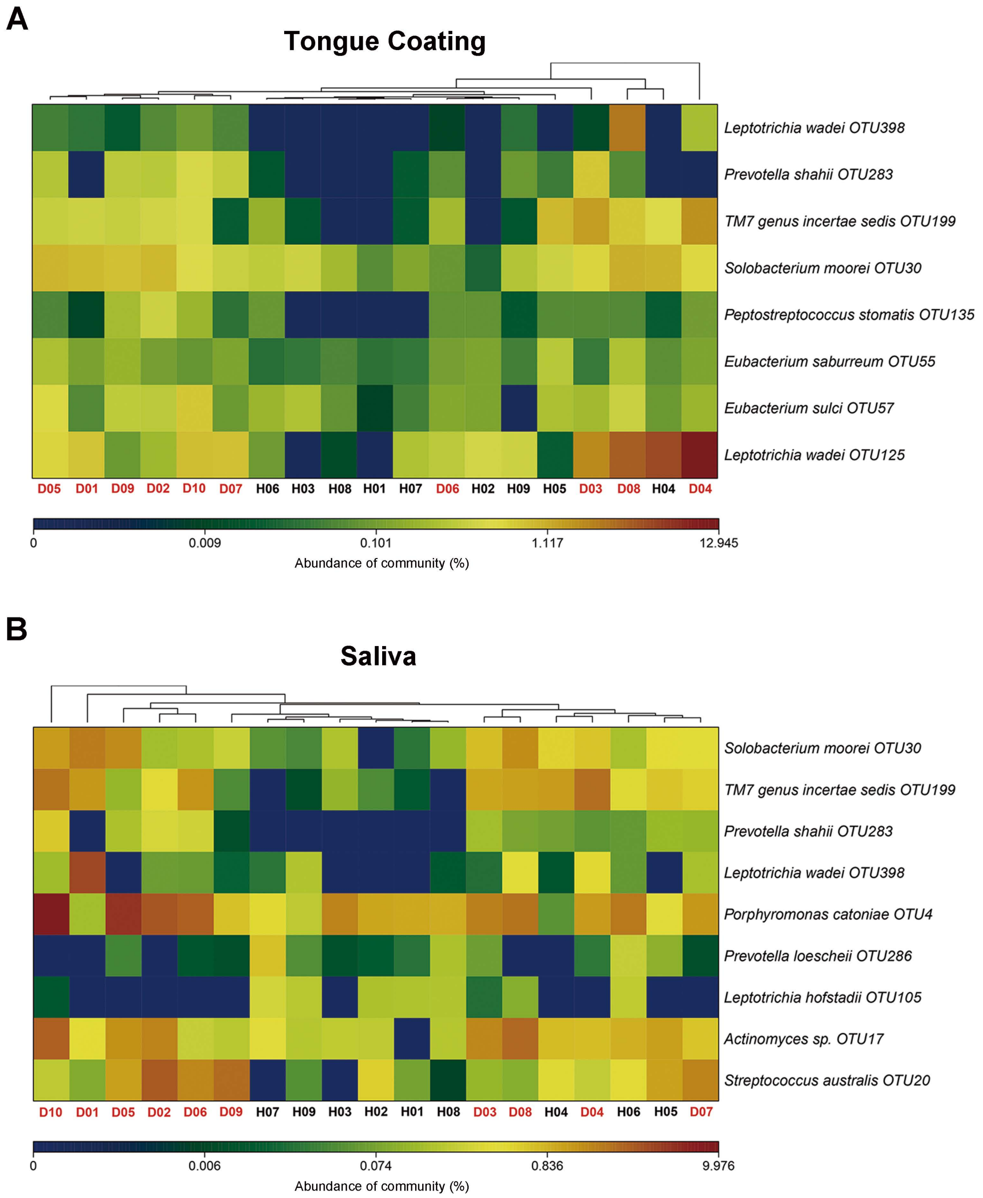

Figure S6. Heatmaps of OTUs with different relative abundances per subject in tongue coating (A) and saliva (B). Samples were clustered based on Manhattan distance. Samples labeled in black represent healthy ones, while samples labeled in red represent halitosis ones. Individual cells of each OTU are color-coded according to relative abundance, with an increasing value from blue to red.

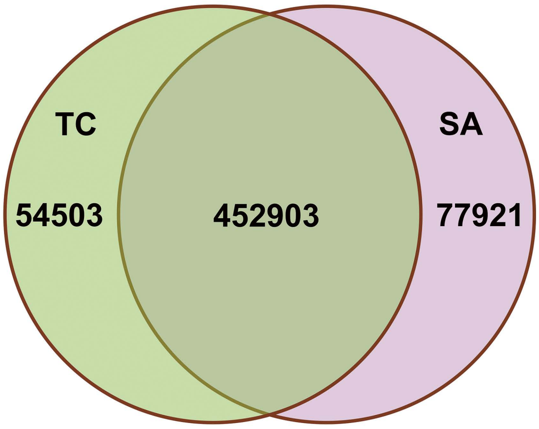

Figure S7. Comparison of oral microbiome gene sets between tongue coating (TC) and saliva samples (SA). Venn diagram of gene sets of tongue coating and saliva samples. The gene number in each group and the overlapping area are shown.

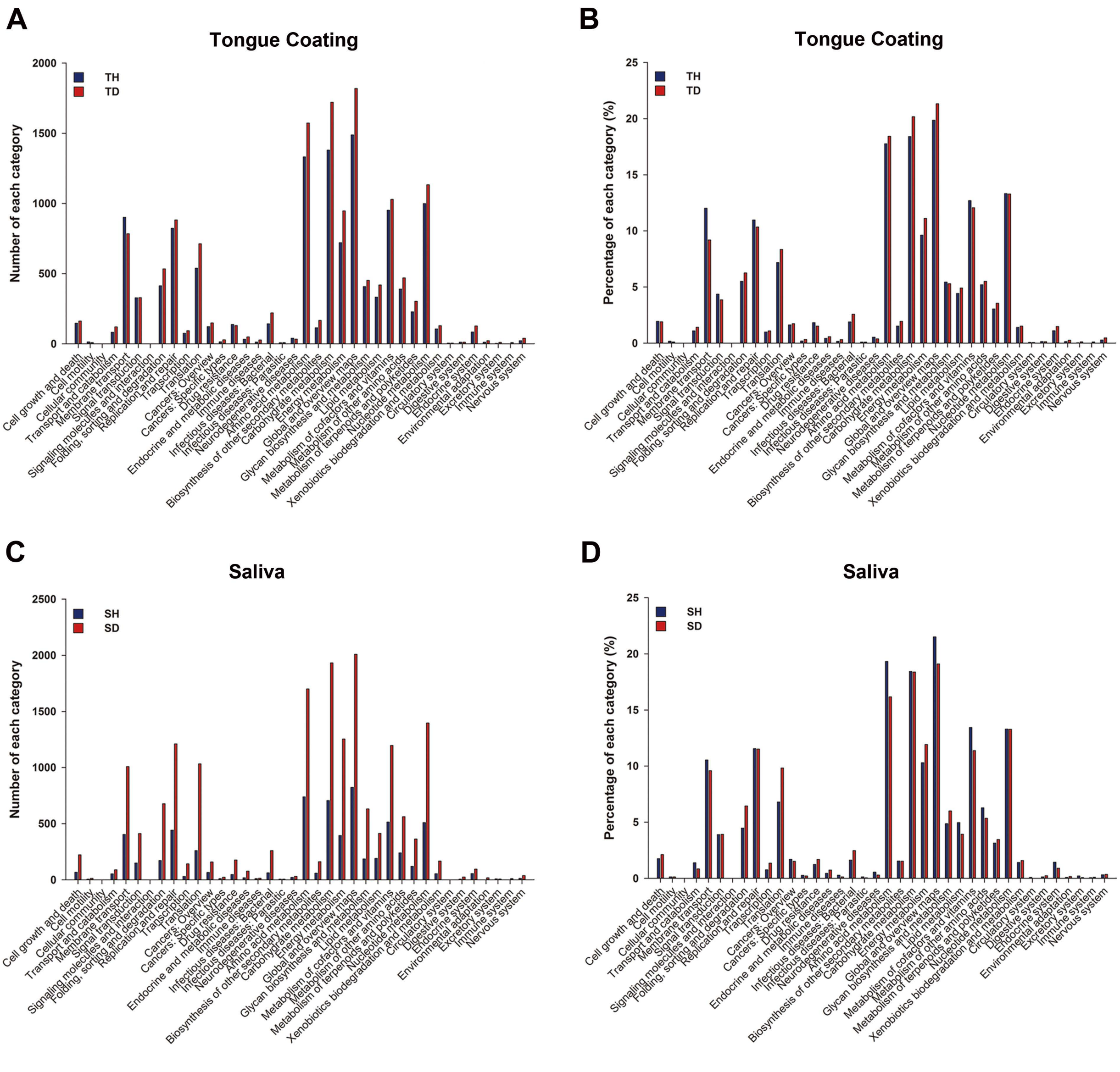

Figure S8. The distribution of KEGG functional categories for differential genes. (**A**) Comparison between the healthy tongue coating (TH) and halitosis tongue coating (TD) differential genes in KEGG functional categories is shown by number. (**B**) Comparison between the healthy tongue coating (TH) and halitosis tongue coating (TD) differential genes in KEGG functional categories is shown by percentage. (**C**) Comparison between the healthy saliva (SH) and halitosis saliva (SD) differential genes in KEGG functional categories is shown by number. (**D**) Comparison between the healthy saliva (SH) and halitosis saliva (SD) differential genes in KEGG functional categories is shown by percentage.

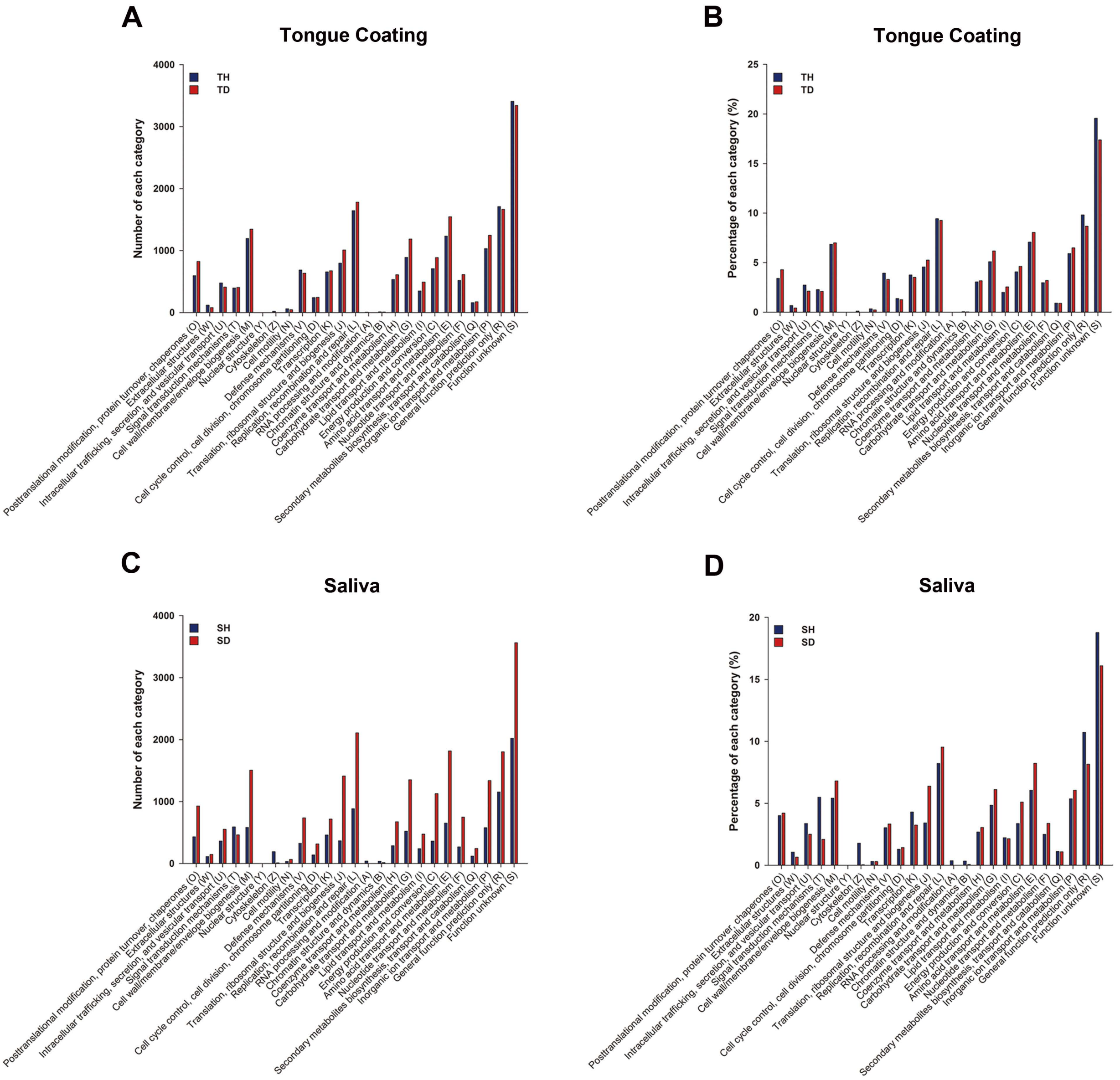

Figure S9. The distribution of eggNOG orthologous groups for differential genes. (**A**) Comparison between the healthy tongue coating (TH) and halitosis tongue coating (TD) differential genes in eggNOG orthologous groups is shown by number. (**B**) Comparison between the healthy tongue coating (TH) and halitosis tongue coating (TD) differential genes in eggNOG orthologous groups is shown by percentage. (**C**) Comparison between the healthy saliva (SH) and halitosis saliva (SD) differential genes in eggNOG orthologous groups is shown by number. (**D**) Comparison between the healthy saliva (SH) and halitosis saliva (SD) differential genes in eggNOG orthologous groups is shown by percentage.

Supplementary Tables

Table S1-1 Basic details of the 20 evaluated subjects

Characteristics	Health (n=9[c])	Halitosis (n=10)
Female (%)	55.56	80
H_2S[a] (mean ± s.d.)	0.01 ± 0.02	4.58 ± 2.37
CH_3SH[a] (mean ± s.d.)	0	1.81 ± 4.84
$(CH_3)_2S$[a] (mean ± s.d.)	0	1.96 ± 5.18
dmft[b] (mean ± s.d.)	2.33 ± 3.04	1.80 ± 1.81
Caries prevalence (%)	0.5556	0.6

[a] $P < 0.05$ (Wilcoxon rank-sum test). H_2S, CH_3SH, $(CH_3)_2S$ were measured by ng $10ml^{-1}$.

[b] decayed-missing-filled teeth.

[c] One healthy subject was excluded because the number of reads of his tongue coating sample were too poor by *16S rRNA* gene pyrosequencing.

Table S1-2 Basic details of the 20 evaluated subjects

ID	Gender (M/F)	Age (years)	H_2S (ng/ml)	CH_3SH (ng/ml)	$(CH_3)_2S$ (ng/ml)	dmft*	dt*	TCA*	TCT*	GI*	DI-S*	Saliva pH	Systemic Diseases (Y/N)
HC**01	F	4	0	0	0	0	0	1	1	1.14	8	7.4	N
HC02	M	4	0	0	0	9	5	2	2	1	5	7	N
HC03	F	4	0	0	0	0	0	2	2	1.06	7	6.8	N
HC04	F	4	0	0	0	2	2	2	2	1.06	10	6.9	N
HC05	M	4	0	0	0	3	3	2	2	1.11	10	7.1	N
HC06	F	4	0	0	0	2	2	2	1	1.11	8	6.9	N
HC07	M	4	0	0	0	5	0	1	1	1.06	13	6.7	N
HC08	F	4	0.02	0	0	0	3	1	1	1	7	7.1	N
HC09	M	4	0.05	0	0	0	0	1	1	1.08	13	7.1	N
HC10	F	4	0	0	0	2	0	2	2	1	6	7.1	N
DIS**01	F	4	1.58	0.49	1.54	0	0	1	1	1.08	9	7.4	N
DIS02	F	4	1.64	15.55	16.62	0	0	2	1	1.03	8	6.9	N
DIS03	F	5	1.85	0.24	1.14	3	4	2	2	1.08	5	7	N
DIS04	F	4	3.24	1.15	0	3	3	2	2	1.06	11	6.9	N
DIS05	M	4	5.97	0.56	0.31	3	3	1	2	1	5	7.3	N
DIS06	F	4	6.03	0.11	0	0	0	2	1	1.08	5	7	N
DIS07	F	4	6.15	0	0	5	2	2	1	1	7	7.4	N
DIS08	F	4	7.49	0.03	0	3	2	2	2	1.22	8	7	N
DIS09	M	4	7.5	0	0	0	0	2	1	1.19	11	7.2	N
DIS10	F	5	4.34	0	0	1	0	1	1	1.17	8	7.1	N

*Abbreviations:

dmft, decayed, missing, filled teeth;

dt, decayed teeth

TCA, Tongue Coating Area;

TCT, Tongue Coating Thickness;

GI, Gingival Index;

DI-S, Debris Index-Simplified.

Criteria were based on Jinyou Bian. in *Preventive Dentistry* (Beijing, 2006).

**HC: healthy samples;

DIS: halitosis samples.

Table S2 Background information and pyrosequencing data of 40 samples

ID	Site	Diagnosis	Raw tag number	Final tag number
TH*01	Tongue coating	Healthy	24,214	11,266
TH02	Tongue coating	Healthy	28,722	11,266
TH03	Tongue coating	Healthy	28,918	12,284
TH04	Tongue coating	Healthy	28,779	9,504
TH05	Tongue coating	Healthy	27,315	9,015
TH06	Tongue coating	Healthy	31,935	12,144
TH07	Tongue coating	Healthy	27,240	9,873
TH08	Tongue coating	Healthy	19,193	8,589
TH09	Tongue coating	Healthy	24,610	11,626
TH10	Tongue coating	Healthy	13,843	4,780
TD*01	Tongue coating	Halitosis	27,128	10,782
TD02	Tongue coating	Halitosis	28,779	10,814
TD03	Tongue coating	Halitosis	27,032	9,017
TD04	Tongue coating	Halitosis	27,416	8,670
TD05	Tongue coating	Halitosis	31,572	11,825
TD06	Tongue coating	Halitosis	23,721	11,061
TD07	Tongue coating	Halitosis	29,612	13,904
TD08	Tongue coating	Halitosis	28,295	9,034
TD09	Tongue coating	Halitosis	23,201	10,476
TD10	Tongue coating	Halitosis	26,779	11,949
SH*01	Saliva	Healthy	23,092	8,019
SH02	Saliva	Healthy	30,502	7,180
SH03	Saliva	Healthy	28,147	11,205
SH04	Saliva	Healthy	28,318	9,583
SH05	Saliva	Healthy	30,057	10,320
SH06	Saliva	Healthy	32,325	10,182
SH07	Saliva	Healthy	39,244	11,814
SH08	Saliva	Healthy	36,669	16,167
SH09	Saliva	Healthy	34,904	12,633
SH10	Saliva	Healthy	30,838	10,661
SD*01	Saliva	Halitosis	30,691	10,037
SD02	Saliva	Halitosis	30,235	7,496
SD03	Saliva	Halitosis	25,292	8,782
SD04	Saliva	Halitosis	21,941	7,396
SD05	Saliva	Halitosis	29,706	10,741
SD06	Saliva	Halitosis	25,994	9,705
SD07	Saliva	Halitosis	33,605	12,175
SD08	Saliva	Halitosis	23,299	7,381
SD09	Saliva	Halitosis	33,545	11,755
SD10	Saliva	Halitosis	30,608	9,027

*TH: healthy tongue coating sample;
TD: tongue coating sample with halitosis;
SH: healthy saliva sample;
SD: saliva sample with halitosis.

Table S3 α diversity estimators of 38 samples

ID	Observed OTUs	CatchAll	Chao1
TH*01	97	237.20	164.57
TH02	98	225.10	138.55
TH03	94	189.30	130.91
TH04	120	219.70	188.44
TH05	118	273.50	169.57
TH06	110	311.60	169.67
TH07	107	225.90	152.00
TH08	66	151.00	89.00
TH09	130	263.90	186.88
TD*01	99	180.30	148.75
TD02	105	238.70	164.91
TD03	176	535.60	268.88
TD04	181	384.20	241.64
TD05	182	531.90	263.95
TD06	96	218.60	129.83
TD07	95	257.80	133.27
TD08	135	392.10	224.00
TD09	120	328.90	202.67
TD10	142	280.00	190.32
SH*01	152	244.70	188.00
SH02	155	482.60	261.07
SH03	170	369.00	223.25
SH04	183	385.60	254.35
SH05	166	340.10	199.56
SH06	266	446.40	321.04
SH07	249	424.20	292.24
SH08	220	355.80	234.23
SH09	238	375.00	276.04
SD*01	162	349.80	211.71
SD02	175	348.00	275.12
SD03	196	328.20	240.00
SD04	202	446.20	281.36
SD05	151	253.10	188.09
SD06	167	231.10	200.00
SD07	183	549.20	229.50
SD08	194	425.70	273.38
SD09	201	393.30	244.64
SD10	230	410.40	294.20

*TH: healthy tongue coating sample;
TD: tongue coating sample with halitosis;
SH: healthy saliva sample;
SD: saliva sample with halitosis.

Table S4-1 The characteristics of hub nodes in co-occurrence network (healthy module)

OTU ID	Taxa	Group	Colour of node	Average relative abundance (%)	Characteristics	References*
327	*Capnocytophaga_leadbetteri*	TH*	Green	0.0047	associated with chorioamionitis	1
70	*Capnocytophaga_gingivalis*	TH	Green	0.043	cause bacteremia, pyogenic arthritis, lung abscess	2-4
166	*Campylobacter_showae*	SH*	Orange	0.0573	cause bacteremia with cholangitis	5
368	*Campylobacter_gracilis*	SH	Orange	0.0032	participate in infections of endodontic origin	6
839	Bacteria	SH	Orange	0.007	-	-
361	Bacteria	SH	Orange	0.0737	-	-
359	Bacteria	SH	Orange	0.1304	-	-
811	*Actinomyces_dentalis*	SH	Orange	0.0051	isolated from pus of a human dental abscess	7
285	*Actinomyces*	SH	Orange	0.0832	cause human infection	8-10
191	Unclassified	SH	Orange	0.1119	-	-
158	Unclassified	SH	Orange	0.0702	-	-
336	*TM7_genus_incertae_sedis*	SH	Orange	0.0611	-	-
61	*Streptococcus_intermedius*	SH	Orange	0.0938	cause purulent infections	11-14
534	*Selenomonas_noxia*	SH	Orange	0.0602	associated with periodontitis	15-17
507	*Selenomonas_infelix*	SH	Orange	0.0227	cause bacteremia	18
159	*Selenomonas_infelix*	SH	Orange	0.008	cause bacteremia	18
195	*Prevotella_oris*	SH	Orange	0.0665	periodontopathogens, coaggregate with Porphyromonas gingivalis	19
63	*Leptotrichia*	SH	Orange	0.4138	cause human infection	20-22
287	*Fusobacterium* sp.	SH	Orange	0.0491	production of volatile sulfur compounds, bacteremia	23,24
67	*Fusobacterium*	SH	Orange	0.0449	cause human infection	25,26
367	Flavobacteriaceae	SH	Orange	0.0211	-	-
754	*Cardiobacterium_valvarum*	SH	Orange	0.002	cause aortic vascular graft infection and Endocarditis	27,28
675	*Cardiobacterium*	SH	Orange	0.0021	cause endocarditis	29
333	Actinomycetales	SH	Orange	0.0061	-	-
478	*Prevotella_saccharolytica*	SH	Orange	0.0021	isolated from the human oral cavity	30
494	Flavobacteriaceae	SH	Orange	0.0161	-	-
538	*Treponema_maltophilum*	SH	Orange	0.0027	isolated from human periodontal lesions	31

*TH: healthy tongue coating sample;
SH: healthy saliva sample.
References please see Table S4-3

Table S4-2 The characteristics of hub nodes in co-occurrence network (halitosis module)

OTU ID	Taxa	Group	Colour of node	relative abundance (%)	Characteristics	References*
408	*Fusobacterium* sp.	TD*	Green	0.002	produce volatile sulfur compounds, cause bacteremia	23,24
395	*Corynebacterium_matruchotii*	TD	Green	0.0166	prevalent in infected root canals, significantly decreased as caries progressed, had higher abundance in subgingival microbiome of smokers	32-34
393	*Capnocytophaga_ochracea*	TD	Green	0.0148	cause severe sepsis, conjunctivitis, cervical abscess	35-37
147	*Capnocytophaga_ochracea*	TD	Green	0.004	cause severe sepsis, conjunctivitis, cervical abscess	35-37
48	*Capnocytophaga*	TD	Green	0.0235	cause local bone infection, bacteremia	38,39
334	*Campylobacter_gracilis*	TD	Green	0.009	participate in infections of endodontic origin	6
359	Bacteria	TD	Green	0.013	-	-
64	Actinomycetales	TD	Green	0.0031	-	-
479	*Actinomyces_massiliensis*	TD	Green	0.0031	isolated from a human blood sample	40
132	*Actinomyces_graevenitzii*	TD	Green	0.1223	associated with lung abscess, bacteremia	41,42
232	*Actinomyces_graevenitzii*	TD	Green	0.0258	associated with lung abscess, bacteremia	41,42
287	*Fusobacterium* sp.	TD	Green	0.0104	production of volatile sulfur compounds, bacteremia	23,24
499	*Actinomyces* sp.	TD	Green	0.004	cause human infection	8-10
218	*Actinomyces* sp.	TD	Green	0.0051	cause human infection	8-10
16	*Actinomyces* sp.	TD	Green	1.535	cause human infection	8-10
191	Unclassified	TD	Green	0.0727	-	-
158	Unclassified	TD	Green	0.0303	-	-
406	*Selenomonas_noxia*	TD	Green	0.0121	associated with periodontitis	15-17
397	*Prevotella_micans*	TD	Green	0.002	isolated from human oral cavity	43
31	*Prevotella_histicola*	TD	Green	2.1943	isolated from human oral cavity	44
105	*Leptotrichia_hofstadii*	TD	Green	0.0752	isolated from healthy human saliva	45
201	*Leptotrichia* sp.	TD	Green	0.0423	cause human infection	20-22
1	*Neisseria_flavescens*	TD	Green	28.9305	cause meningitis, septicaemia, infective endocarditis, pneumonia	46-49
123	*Prevotella* sp.	SD*	Orange	0.3928	cause human infection	50
6	*Granulicatella_adiacens*	SD	Orange	2.3351	cause septic arthritis, endocarditis	51,52
9	*Gemella_sanguinis*	SD	Orange	1.2647	cause endocarditis, prosthetic joint infection, prosthetic valve endocarditis	53-55
232	*Actinomyces_graevenitzii*	SD	Orange	0.0399	associated with lung abscess, bacteremia	41,42

*TD: tongue coating sample with halitosis;
SD: saliva sample with halitosis.
References please see Table S4-3

Table S4-3 References

1 Mekouar, H. *et al.* Capnocytophaga species and perinatal infections: case report and review of the literature. *Acta clinica Belgica* **67**, 42-45, doi:10.1179/ACB.67.1.2062626 (2012).

2 Fukuoka, K. *et al.* [A case report of lung abscess caused by Capnocytophaga gingivalis infection]. *Kansenshogaku zasshi. The Journal of the Japanese Association for Infectious Diseases* **74**, 594-597 (2000).

3 Mantadakis, E., Danilatou, V., Christidou, A., Stiakaki, E. & Kalmanti, M. Capnocytophaga gingivalis bacteremia detected only on quantitative blood cultures in a child with leukemia. *The Pediatric infectious disease journal* **22**, 202-204 (2003).

4 Rodgers, G. L., Mortensen, J. E. & Goldsmith, D. P. Pyogenic arthritis caused by capnocytophaga gingivalis in an immunocompetent three-year-old male. *Journal of clinical rheumatology : practical reports on rheumatic & musculoskeletal diseases* **7**, 265-267 (2001).

5 Suzuki, J. *et al.* Campylobacter showae bacteremia with cholangitis. *Journal of infection and chemotherapy : official journal of the Japan Society of Chemotherapy* **19**, 960-963, doi:10.1007/s10156-012-0524-2 (2013).

6 Siqueira, J. F., Jr. & Rocas, I. N. Campylobacter gracilis and Campylobacter rectus in primary endodontic infections. *International endodontic journal* **36**, 174-180 (2003).

7 Hall, V., Collins, M. D., Lawson, P. A., Falsen, E. & Duerden, B. I. Actinomyces dentalis sp. nov., from a human dental abscess. *International journal of systematic and evolutionary microbiology* **55**, 427-431, doi:10.1099/ijs.0.63376-0 (2005).

8 Govindarajah, N., Hameed, W., Middleton, S. & Booth, M. Actinomyces infection causing acute right iliac fossa pain. *BMJ case reports* **2014**, doi:10.1136/bcr-2014-204559 (2014).

9 Masata, J., Dundr, P. & Martan, A. Actinomyces infection appearing five years after trocar-guided transvaginal mesh prolapse repair. *International urogynecology journal* **25**, 993-996, doi:10.1007/s00192-013-2304-7 (2014).

10 Pakish, J. B. & West, L. Actinomyces bacteremia in association with tubo-ovarian abscesses and hysteroscopic sterilization. *Obstetrics and gynecology* **124**, 451-453, doi:10.1097/AOG.0000000000000382 (2014).

11 Livingston, L. V. & Perez-Colon, E. Streptococcus intermedius Bacteremia and Liver Abscess following a Routine Dental Cleaning. *Case reports in infectious diseases* **2014**, 954046, doi:10.1155/2014/954046 (2014).

12 Mishra, A. K. & Fournier, P. E. The role of Streptococcus intermedius in brain abscess. *European journal of clinical microbiology & infectious diseases : official publication of the European Society of Clinical Microbiology* **32**, 477-483, doi:10.1007/s10096-012-1782-8 (2013).

13 Presnell, L., Maeda, K., Griffin, M. & Axelrod, D. A child with purulent pericarditis and Streptococcus intermedius in the presence of a pericardial teratoma: an unusual presentation. *The Journal of thoracic and cardiovascular surgery* **147**, e23-24, doi:10.1016/j.jtcvs.2013.11.025 (2014).

14 Trabue, C., Pearman, R. & Doering, T. Pyogenic brain and lung abscesses due to Streptococcus intermedius. *Journal of general internal medicine* **29**, 407, doi:10.1007/s11606-013-2565-3 (2014).

15 Colombo, A. P. *et al.* Comparisons of subgingival microbial profiles of refractory periodontitis, severe periodontitis, and periodontal health using the human oral microbe identification microarray. *Journal of periodontology* **80**, 1421-1432,

doi:10.1902/jop.2009.090185 (2009).

16 Faveri, M. *et al.* Microbiological diversity of generalized aggressive periodontitis by 16S rRNA clonal analysis. *Oral microbiology and immunology* **23**, 112-118, doi:10.1111/j.1399-302X.2007.00397.x (2008).

17 Lopez, R., Dahlen, G., Retamales, C. & Baelum, V. Clustering of subgingival microbial species in adolescents with periodontitis. *European journal of oral sciences* **119**, 141-150, doi:10.1111/j.1600-0722.2011.00808.x (2011).

18 Bisiaux-Salauze, B., Perez, C., Sebald, M. & Petit, J. C. Bacteremias caused by Selenomonas artemidis and Selenomonas infelix. *Journal of clinical microbiology* **28**, 140-142 (1990).

19 Sato, T. & Nakazawa, F. Coaggregation between Prevotella oris and Porphyromonas gingivalis. *Journal of microbiology, immunology, and infection = Wei mian yu gan ran za zhi* **47**, 182-186, doi:10.1016/j.jmii.2012.09.005 (2014).

20 Cooreman, S. *et al.* Bacteraemia caused by Leptotrichia trevisanii in a neutropenic patient. *Anaerobe* **17**, 1-3, doi:10.1016/j.anaerobe.2010.12.002 (2011).

21 Couturier, M. R., Slechta, E. S., Goulston, C., Fisher, M. A. & Hanson, K. E. Leptotrichia bacteremia in patients receiving high-dose chemotherapy. *Journal of clinical microbiology* **50**, 1228-1232, doi:10.1128/JCM.05926-11 (2012).

22 Eribe, E. R. & Olsen, I. Leptotrichia species in human infections. *Anaerobe* **14**, 131-137, doi:10.1016/j.anaerobe.2008.04.004 (2008).

23 Claesson, R., Edlund, M. B., Persson, S. & Carlsson, J. Production of volatile sulfur compounds by various Fusobacterium species. *Oral microbiology and immunology* **5**, 137-142 (1990).

24 O'Grady, L. R. & Ralph, E. D. Anaerobic meningitis and bacteremia caused by Fusobacterium species. *American journal of diseases of children* **130**, 871-873 (1976).

25 Buelow, B. D., Lambert, J. M. & Gill, R. M. Fusobacterium liver abscess. *Case reports in gastroenterology* **7**, 482-486, doi:10.1159/000356821 (2013).

26 Pett, E., Saeed, K. & Dryden, M. Fusobacterium species infections: clinical spectrum and outcomes at a district general hospital. *Infection* **42**, 363-370, doi:10.1007/s15010-013-0564-2 (2014).

27 Bothelo, E. *et al.* Endocarditis caused by Cardiobacterium valvarum. *Journal of clinical microbiology* **44**, 657-658, doi:10.1128/JCM.44.2.657-658.2006 (2006).

28 Hagiya, H. *et al.* Aortic vascular graft infection caused by Cardiobacterium valvarum: a case report. *Journal of infection and chemotherapy : official journal of the Japan Society of Chemotherapy* **20**, 804-809, doi:10.1016/j.jiac.2014.07.008 (2014).

29 Walkty, A. Cardiobacterium hominis endocarditis: A case report and review of the literature. *The Canadian journal of infectious diseases & medical microbiology = Journal canadien des maladies infectieuses et de la microbiologie medicale / AMMI Canada* **16**, 293-297 (2005).

30 Downes, J., Tanner, A. C., Dewhirst, F. E. & Wade, W. G. Prevotella saccharolytica sp. nov., isolated from the human oral cavity. *International journal of systematic and evolutionary microbiology* **60**, 2458-2461, doi:10.1099/ijs.0.014720-0 (2010).

31 Wyss, C., Choi, B. K., Schupbach, P., Guggenheim, B. & Gobel, U. B. Treponema maltophilum sp. nov., a small oral spirochete isolated from human periodontal lesions. *International journal of systematic bacteriology* **46**, 745-752 (1996).

32 Gross, E. L. *et al.* Bacterial 16S sequence analysis of severe caries in young permanent teeth. *Journal of clinical microbiology* **48**, 4121-4128, doi:10.1128/JCM.01232-10 (2010).

33 Moon, J. H., Lee, J. H. & Lee, J. Y. Subgingival microbiome in smokers and non-smokers in Korean chronic periodontitis patients. *Molecular oral microbiology*, doi:10.1111/omi.12086 (2014).

34 Siqueira, J. F., Jr., Rocas, I. N., Souto, R., de Uzeda, M. & Colombo, A. P. Checkerboard DNA-DNA hybridization analysis of endodontic infections. *Oral surgery, oral medicine, oral pathology, oral radiology, and endodontics* **89**, 744-748 (2000).

35 Desai, S. S., Harrison, R. A. & Murphy, M. D. Capnocytophaga ochracea causing severe sepsis and purpura fulminans in an immunocompetent patient. *The Journal of infection* **54**, e107-109, doi:10.1016/j.jinf.2006.06.014 (2007).

36 Henzen, C. & Streit, E. [Cervical abscess caused by Capnocytophaga ochracea]. *Schweizerische medizinische Wochenschrift* **123**, 1165-1168 (1993).

37 Wasserman, D., Asbell, P. A., Friedman, A. J. & Bottone, E. J. Capnocytophaga ochracea chronic blepharoconjunctivitis. *Cornea* **14**, 533-535 (1995).

38 Piau, C., Arvieux, C., Bonnaure-Mallet, M. & Jolivet-Gougeon, A. Capnocytophaga spp. involvement in bone infections: a review. *Int J Antimicrob Agents* **41**, 509-515, doi:10.1016/j.ijantimicag.2013.03.001 (2013).

39 Pokroy-Shapira, E., Shiber, S. & Molad, Y. Capnocytophaga bacteraemia following rituximab treatment. *BMJ case reports* **2012**, doi:10.1136/bcr-2012-006224 (2012).

40 Renvoise, A., Raoult, D. & Roux, V. Actinomyces massiliensis sp. nov., isolated from a patient blood culture. *International journal of systematic and evolutionary microbiology* **59**, 540-544, doi:10.1099/ijs.0.001503-0 (2009).

41 Hwang, S. S. *et al.* Actinomyces graevenitzii bacteremia in a patient with alcoholic liver cirrhosis. *Anaerobe* **17**, 87-89, doi:10.1016/j.anaerobe.2011.03.002 (2011).

42 Nagaoka, K. *et al.* Multiple lung abscesses caused by Actinomyces graevenitzii mimicking acute pulmonary coccidioidomycosis. *Journal of clinical microbiology* **50**, 3125-3128, doi:10.1128/JCM.00761-12 (2012).

43 Downes, J., Liu, M., Kononen, E. & Wade, W. G. Prevotella micans sp. nov., isolated from the human oral cavity. *International journal of systematic and evolutionary microbiology* **59**, 771-774, doi:10.1099/ijs.0.002337-0 (2009).

44 Downes, J., Hooper, S. J., Wilson, M. J. & Wade, W. G. Prevotella histicola sp. nov., isolated from the human oral cavity. *International journal of systematic and evolutionary microbiology* **58**, 1788-1791, doi:10.1099/ijs.0.65656-0 (2008).

45 Eribe, E. R. *et al.* Genetic diversity of Leptotrichia and description of Leptotrichia goodfellowii sp. nov., Leptotrichia hofstadii sp. nov., Leptotrichia shahii sp. nov. and Leptotrichia wadei sp. nov. *International journal of systematic and evolutionary microbiology* **54**, 583-592 (2004).

46 Huang, L. *et al.* Necrotizing pneumonia and empyema caused by Neisseria flavescens infection. *Journal of thoracic disease* **6**, 553-557, doi:10.3978/j.issn.2072-1439.2014.02.16 (2014).

47 Prentice, A. W. Neisseria flavescens as a cause of meningitis. *Lancet* **272**, 613-614 (1957).

48 Sinave, C. P. & Ratzan, K. R. Infective endocarditis caused by Neisseria flavescens. *The American journal of medicine* **82**, 163-164 (1987).

49 Wertlake, P. T. & Williams, T. W., Jr. Septicaemia caused by Neisseria flavescens. *Journal of clinical pathology* **21**, 437-439 (1968).
50 Amissah-Arthur, K. N., Farooq, T. A., Dhillon, N., Cunliffe, I. A. & Bansal, A. Rare case of post-cataract-surgery Prevotella endophthalmitis diagnosed by polymerase chain reaction DNA sequencing. *Journal of cataract and refractive surgery* **39**, 463-466, doi:10.1016/j.jcrs.2012.12.021 (2013).
51 Hepburn, M. J., Fraser, S. L., Rennie, T. A., Singleton, C. M. & Delgado, B., Jr. Septic arthritis caused by Granulicatella adiacens: diagnosis by inoculation of synovial fluid into blood culture bottles. *Rheumatology international* **23**, 255-257, doi:10.1007/s00296-003-0305-4 (2003).
52 Perkins, A. *et al.* A case of endocarditis due to Granulicatella adiacens. *Clinical microbiology and infection : the official publication of the European Society of Clinical Microbiology and Infectious Diseases* **9**, 576-577 (2003).
53 Gundre, P., Pascal, W., Abrol, S., Kupfer, Y. & Tessler, S. Prosthetic valve endocarditis caused by Gemella sanguinis: a consequence of persistent dental infection. *The American journal of the medical sciences* **341**, 512-513, doi:10.1097/MAJ.0b013e31821389f0 (2011).
54 Leung, D. T., Davis, E. M., Qian, Q. & Gold, H. S. First report of prosthetic joint infection by Gemella sanguinis and associated "pseudosatelliting" phenomenon on culture. *Journal of clinical microbiology* **49**, 3395-3397, doi:10.1128/JCM.01050-11 (2011).
55 Yang, C. H. & Tsai, K. T. Gemella sanguinis endocarditis: first case report in Taiwan and review of the literature. *Journal of the Formosan Medical Association = Taiwan yi zhi* **113**, 562-565, doi:10.1016/j.jfma.2012.02.012 (2014).

Table S5 Statistics of metagenomic sequencing of samples

Sample	Clean PE-reads	Human PE-reads	Microbial PE-reads	Human DNA contamination
TH*	22,882,448	2,068,573	20,813,875	9.04%
TD*	23,364,951	668,237	22,696,714	2.86%
SH*	31,314,275	20,942,987	10,371,288	66.88%
SD*	23,349,421	12,174,388	11,175,033	52.14%

*TH: healthy tongue coating sample;
TD: tongue coating sample with halitosis;
SH: healthy saliva sample;
SD: saliva sample with halitosis;

Table S6 Gene count of samples

Sample_id	Gene count
TH*	436,024
TD*	448,263
SH*	445,829
SD*	478,922
TC*	507,406
SA*	530,824
Total	585,327

*TH: healthy tongue coating sample;
TD: tongue coating sample with halitosis;
SH: healthy saliva sample;
SD: saliva sample with halitosis;
TC: tongue coating samples;
SA: saliva samples.

Table S7-1 Enrichment analysis of KEGG ontology (enrichment analysis of level 2)

Level 1	Level 2	Total (296279)	Gene_TH*_number (7497)	Gene_TD*_number (8532)	Gene_SH*_number (3825)	Gene_SD*_number (10515)	Total_percent	Gene_TH_percent	Gene_TD_percent	Gene_SH_percent	Gene_SD_percent	Gene_TH_pvalue	Gene_TH_qvalue	Gene_TD_pvalue	Gene_TD_qvalue	Gene_SH_pvalue	Gene_SH_qvalue	Gene_SD_pvalue	Gene_SD_qvalue
Cellular Processes	Cell growth and death	5328	146	162	67	222	0.017983	0.019474	0.018987	0.017516	0.021113	0.173093	0.674617	0.250168	0.912403	0.603362	1	0.008861	0.092229
Cellular Processes	Cell motility	361	13	9	5	13	0.001218	0.001734	0.001055	0.001307	0.001236	0.131313	0.554432	0.713715	1	0.498948	1	0.517771	1
Cellular Processes	Cellular community	13	1	1	0	1	4.39E-05	0.000133	0.000117	0	9.51E-05	0.283366	0.936341	0.316048	0.980393	1	1	0.374853	1
Cellular Processes	Transport and catabolism	3358	82	120	53	89	0.011334	0.010938	0.014065	0.013856	0.008464	0.643871	1	0.010723	0.09588	0.082936	0.38201	0.998628	1
Environmental Information Processing	Membrane transport	28505	901	784	403	1008	0.09621	0.120181	0.091889	0.105359	0.095863	2.94E-12	2.23E-10	0.918721	1	0.029663	0.173416	0.553912	1
Environmental Information Processing	Signal transduction	13104	327	329	149	412	0.044229	0.043617	0.038561	0.038954	0.039182	0.610872	1	0.996069	1	0.951299	1	0.995705	1
Environmental Information Processing	Signaling molecules and interaction	10	1	1	0	1	3.38E-05	0.000133	0.000117	0	9.51E-05	0.226089	0.85914	0.253384	0.912403	1	1	0.303271	0.960651
Genetic Information Processing	Folding, sorting and degradation	18273	413	534	171	678	0.061675	0.055089	0.062588	0.044706	0.064479	0.993057	1	0.367516	1	0.999998	1	0.116197	0.504629
Genetic Information Processing	Replication and repair	27677	822	882	442	1211	0.093415	0.109644	0.103376	0.115556	0.115169	9.62E-07	3.65E-05	0.000825	0.015678	2.48E-06	7.55E-05	2.1E-14	3.19E-12
Genetic Information Processing	Transcription	4767	74	93	29	142	0.01609	0.009871	0.0109	0.007582	0.013505	0.999999	1	0.999982	1	0.999999	1	0.987232	1
Genetic Information Processing	Translation	29386	538	712	260	1033	0.099184	0.071762	0.083451	0.067974	0.098241	1	1	1	1	1	1	0.633956	1
Human Diseases	Cancers: Overview	4045	122	148	65	159	0.013653	0.016273	0.017346	0.016993	0.015121	0.029282	0.173416	0.002274	0.032925	0.046149	0.250523	0.101854	0.455348
Human Diseases	Cancers: Specific types	794	14	28	10	22	0.00268	0.001867	0.003282	0.002614	0.002092	0.938913	1	0.161722	0.646887	0.574203	1	0.904225	1
Human Diseases	Drug resistance	4123	137	130	47	177	0.013916	0.018274	0.015237	0.012288	0.016833	0.001013	0.017102	0.156257	0.641922	0.824468	1	0.006331	0.074025
Human Diseases	Endocrine and metabolic diseases	2097	32	49	17	78	0.007078	0.004268	0.005743	0.004444	0.007418	0.999373	1	0.944138	1	0.985263	1	0.351939	1
Human Diseases	Immune diseases	674	12	27	11	14	0.002275	0.001601	0.003165	0.002876	0.001331	0.920178	1	0.056465	0.276862	0.258114	0.912403	0.989964	1
Human Diseases	Infectious diseases: Bacterial	6615	142	220	62	260	0.022327	0.018941	0.025785	0.016209	0.024727	0.981739	1	0.017053	0.1296	0.997016	1	0.049913	0.261615
Human Diseases	Infectious diseases: Parasitic	283	7	8	5	5	0.000955	0.000934	0.000938	0.001307	0.000476	0.576238	1	0.570097	1	0.303363	0.960651	0.973575	1
Human Diseases	Neurodegenerative diseases	1465	40	33	21	31	0.004945	0.005335	0.003868	0.00549	0.002948	0.3347	0.997536	0.939903	1	0.344493	1	0.999454	1
Metabolism	Amino acid metabolism	52036	1332	1573	739	1700	0.175632	0.177671	0.184365	0.193203	0.161674	0.323777	0.984283	0.016731	0.1296	0.002383	0.032925	0.999948	1
Metabolism	Biosynthesis of other secondary metabolites	4839	114	166	59	161	0.016333	0.015206	0.019456	0.015425	0.015311	0.794233	1	0.013331	0.112576	0.689684	1	0.809971	1
Metabolism	Carbohydrate metabolism	60295	1380	1721	705	1933	0.203508	0.184074	0.201711	0.184314	0.183833	0.999991	1	0.666313	1	0.998737	1	1	1
Metabolism	Energy metabolism	38117	720	947	394	1254	0.128652	0.096038	0.110994	0.103007	0.119258	1	1	1	1	1	1	0.998518	1
Metabolism	Global and overview maps	63754	1489	1819	823	2009	0.215182	0.198613	0.213197	0.215163	0.19106	0.999829	1	0.678751	1	0.507581	1	1	1
Metabolism	Glycan biosynthesis and metabolism	14328	407	452	186	631	0.04836	0.054288	0.052977	0.048627	0.06001	0.009102	0.092229	0.024355	0.160958	0.479728	1	2.31E-08	1.17E-06
Metabolism	Lipid metabolism	13248	332	419	190	413	0.044715	0.044284	0.049109	0.049673	0.039277	0.580454	1	0.02585	0.163719	0.074652	0.354597	0.997591	1
Metabolism	Metabolism of cofactors and vitamins	33194	952	1029	514	1197	0.112036	0.126984	0.120605	0.134379	0.113837	2.37E-05	0.000514	0.006093	0.074025	9.56E-06	0.000242	0.279952	0.936341
Metabolism	Metabolism of other amino acids	16250	390	470	240	563	0.054847	0.052021	0.055087	0.062745	0.053543	0.867829	1	0.467605	1	0.01832	0.132605	0.731145	1
Metabolism	Metabolism of terpenoids and polyketides	9210	228	303	120	363	0.031086	0.030412	0.035513	0.031373	0.034522	0.642579	1	0.010178	0.09588	0.471983	1	0.022003	0.152022
Metabolism	Nucleotide metabolism	41170	999	1134	509	1397	0.138957	0.133253	0.132911	0.133072	0.132858	0.928887	1	0.951585	1	0.860734	1	0.968798	1
Metabolism	Xenobiotics biodegradation and metabolism	4840	105	129	54	167	0.016336	0.014006	0.01512	0.014118	0.015882	0.954037	1	0.826765	1	0.877253	1	0.656612	1
Organismal Systems	Circulatory system	218	5	5	3	1	0.000736	0.000667	0.000586	0.000784	9.51E-05	0.64811	1	0.754549	1	0.535301	1	0.999622	1
Organismal Systems	Digestive system	618	11	11	5	25	0.002086	0.001467	0.001289	0.001307	0.002378	0.912405	1	0.968406	1	0.900856	1	0.280362	0.936341
Organismal Systems	Endocrine system	4382	83	127	55	95	0.01479	0.011071	0.014885	0.014379	0.009035	0.997892	1	0.483191	1	0.602424	1	1	1
Organismal Systems	Environmental adaptation	697	11	22	3	17	0.002353	0.001467	0.002579	0.000784	0.001617	0.965577	1	0.361111	1	0.994024	1	0.960778	1
Organismal Systems	Excretory system	559	3	10	8	7	0.001887	0.0004	0.001172	0.002092	0.000666	0.999928	1	0.960952	1	0.433785	1	0.999763	1
Organismal Systems	Immune system	158	1	9	2	10	0.000533	0.000133	0.001055	0.000523	0.000951	0.982587	1	0.040166	0.226119	0.606525	1	0.056112	0.276862
Organismal Systems	Nervous system	1473	21	40	12	37	0.004972	0.002801	0.004688	0.003137	0.003519	0.998717	1	0.668699	1	0.966756	1	0.990048	1

*TH: healthy tongue coating sample;
TD: tongue coating sample with halitosis;
SH: healthy saliva sample;
SD: saliva sample with halitosis.

Table S7-2 Enrichment analysis of KEGG ontology (enrichment analysis of level 3)

Level 1	Level 2	Level 3 (pathway)	Total_number	Total_percent	Gene_TH*_number	Gene_TH_percent	Gene_TH_pvalue	Gene_TH_qvalue	Gene_TD*_number	Gene_TD_percent	Gene_TD_pvalue	Gene_TD_qvalue	Gene_SH*_number	Gene_SH_percent	Gene_SH_pvalue	Gene_SH_qvalue	Gene_SD*_number	Gene_SD_percent	Gene_SD_pvalue	Gene_SD_qvalue
Cellular Processes	Cell growth and death	Apoptosis	47	0.0001586	1	0.000133	0.700212	1	1	0.0001172	0.7467672	1	2	0.000523	0.123272	0.569397	7	0.000666	0.001279	0.021737
Cellular Processes	Cell growth and death	Cell cycle - Caulobacter	5137	0.0173384	141	0.018808	0.172573	0.69004	156	0.0182841	0.2595957	0.6865623	62	0.016209	0.72184	1	209	0.019876	0.024961	0.159124
Cellular Processes	Cell motility	Bacterial chemotaxis	200	0.000675	11	0.001467	0.013607	0.143952	6	0.0007032	0.51684	0.9809054	4	0.001046	0.259467	0.867872	8	0.000761	0.416144	1
Cellular Processes	Transport and catabolism	Lysosome	806	0.0027204	15	0.002001	0.912635	1	32	0.0037506	0.0453695	0.2178964	9	0.002353	0.712902	1	37	0.003519	0.070316	0.341535
Cellular Processes	Transport and catabolism	Peroxisome	2548	0.0086	67	0.008937	0.391659	1	88	0.0103141	0.0494516	0.2208838	44	0.011503	0.035227	0.341701	52	0.004945	0.999997	1
Environmental Information Processing	Membrane transport	ABC transporters	18417	0.062161	588	0.078431	6.65E-09	6.68E-07	522	0.0611814	0.6545942	1	276	0.072157	0.006305	0.101935	625	0.059439	0.884978	1
Environmental Information Processing	Membrane transport	Bacterial secretion system	6296	0.0212502	216	0.028812	6.94E-06	0.000232	191	0.0223863	0.2400588	0.6632295	87	0.022745	0.274166	0.901494	263	0.025012	0.004269	0.045833
Environmental Information Processing	Signal transduction	AMPK signaling pathway	978	0.0033009	9	0.0012	0.999924	1	40	0.0046882	0.0188552	0.1403663	9	0.002353	0.883852	1	32	0.003043	0.704354	1
Environmental Information Processing	Signal transduction	MAPK signaling pathway - yeast	78	0.0002633	5	0.000667	0.048009	0.344633	4	0.0004688	0.1875486	0.579958	0	0	1	1	1	9.51E-05	0.94033	1
Genetic Information Processing	Folding, sorting and degradation	Protein processing in endoplasmic reticulum	142	0.0004793	2	0.000267	0.876956	1	9	0.0010549	0.0222828	0.1410703	1	0.000261	0.842072	1	7	0.000666	0.240999	0.774103
Genetic Information Processing	Folding, sorting and degradation	Sulfur relay system	1614	0.0054476	55	0.007336	0.018253	0.181243	64	0.0075012	0.0075105	0.0838671	19	0.004967	0.687999	1	75	0.007133	0.012441	0.095462
Genetic Information Processing	Replication and repair	Base excision repair	5244	0.0176995	167	0.022276	0.001854	0.023295	180	0.021097	0.0101799	0.093007	84	0.021961	0.028616	0.309507	222	0.021113	0.004635	0.047282
Genetic Information Processing	Replication and repair	DNA replication	9610	0.0324356	275	0.036681	0.020739	0.181243	296	0.0346929	0.1229667	0.4119384	164	0.042876	0.000257	0.012448	415	0.039467	3.19E-05	0.000815
Genetic Information Processing	Replication and repair	Homologous recombination	12344	0.0416634	376	0.050153	0.00016	0.004027	399	0.0467651	0.0098735	0.093007	214	0.055948	1.26E-05	0.001219	522	0.049643	2.68E-05	0.000781
Genetic Information Processing	Replication and repair	Mismatch repair	10902	0.0367964	349	0.046552	6.66E-06	0.000232	370	0.0433662	0.0007708	0.0221318	182	0.047582	0.000348	0.013508	517	0.049168	3.26E-11	3.33E-09
Genetic Information Processing	Replication and repair	Nucleotide excision repair	10920	0.0368572	289	0.038549	0.223543	0.7574	334	0.0391467	0.1339215	0.4406743	153	0.04	0.159733	0.673655	443	0.04213	0.002232	0.035022
Genetic Information Processing	Transcription	Basal transcription factors	4	1.35E-05	0	0	1	1	2	0.0002344	0.0047862	0.0601264	0	0	1	1	2	0.00019	0.007204	0.0668
Human Diseases	Cancers: Overview	MicroRNAs in cancer	366	0.0012353	48	0.006403	2.52E-20	5.07E-18	39	0.004571	4.149E-12	4.17E-10	27	0.007059	7.57E-13	1.47E-10	40	0.003804	4.88E-10	3.32E-08
Human Diseases	Cancers: Specific types	Prostate cancer	120	0.000405	1	0.000133	0.953864	1	8	0.0009376	0.0231608	0.1410703	1	0.000261	0.789784	1	6	0.000571	0.25424	0.774103
Human Diseases	Drug resistance	beta-Lactam resistance	4123	0.0139159	137	0.018274	0.001013	0.015657	130	0.0152368	0.1562572	0.4985349	47	0.012288	0.824468	1	177	0.016833	0.006331	0.061502
Human Diseases	Immune diseases	Primary immunodeficiency	542	0.0018294	11	0.001467	0.808284	1	24	0.0028129	0.0269068	0.1523219	7	0.00183	0.550987	1	12	0.001141	0.971249	1
Human Diseases	Infectious diseases: Bacterial	Bacterial invasion of epithelial cells	69	0.0002329	8	0.001067	0.000355	0.00792	0	0	1	1	1	0.000261	0.592088	1	0	0	1	1
Human Diseases	Infectious diseases: Bacterial	Epithelial cell signaling in Helicobacter pylori infection	726	0.0024504	25	0.003335	0.078283	0.433314	21	0.0024613	0.5221735	0.9809054	9	0.002353	0.593682	1	39	0.003709	0.007821	0.069371
Human Diseases	Infectious diseases: Bacterial	Pertussis	173	0.0005839	11	0.001467	0.00485	0.057342	55	0.0064463	3.336E-41	6.706E-39	1	0.000261	0.894459	1	39	0.003709	2.21E-20	4.5E-18
Human Diseases	Infectious diseases: Bacterial	Staphylococcus aureus infection	311	0.0010497	27	0.003601	4.05E-08	2.72E-06	9	0.0010549	0.5404436	0.9965978	12	0.003137	0.000851	0.023591	21	0.001997	0.004096	0.045833
Human Diseases	Infectious diseases: Bacterial	Vibrio cholerae infection	10	3.375E-05	1	0.000133	0.226089	0.7574	1	0.0001172	0.2533838	0.680805	3	0.000784	0.000241	0.012448	0	0	1	1
Human Diseases	Infectious diseases: Parasitic	Amoebiasis	25	8.438E-05	2	0.000267	0.13112	0.628663	1	0.0001172	0.5183458	0.9809054	2	0.000523	0.041079	0.346489	3	0.000285	0.057469	0.29309
Human Diseases	Neurodegenerative diseases	Amyotrophic lateral sclerosis (ALS)	228	0.0007695	15	0.002001	0.000795	0.013311	8	0.0009376	0.3359048	0.7760559	4	0.001046	0.340022	0.997506	1	9.51E-05	0.999737	1
Metabolism	Amino acid metabolism	Cysteine and methionine metabolism	8849	0.0298671	299	0.039883	4.82E-07	2.42E-05	292	0.0342241	0.0100168	0.093007	135	0.035294	0.028717	0.309507	376	0.035758	0.00024	0.004894
Metabolism	Amino acid metabolism	Histidine metabolism	1949	0.0065783	48	0.006403	0.595513	1	95	0.0111346	8.685E-07	5.819E-05	43	0.011242	0.000664	0.021472	70	0.006657	0.47638	1
Metabolism	Amino acid metabolism	Lysine biosynthesis	5653	0.01908	183	0.02441	0.00057	0.010425	180	0.021097	0.0913079	0.32773	82	0.021438	0.15542	0.673641	219	0.020827	0.098448	0.436594
Metabolism	Amino acid metabolism	Phenylalanine metabolism	1059	0.0035743	38	0.005069	0.022205	0.185964	49	0.0057431	0.0010192	0.0256064	11	0.002876	0.803743	1	53	0.00504	0.008894	0.072575
Metabolism	Amino acid metabolism	Tyrosine metabolism	2170	0.0073242	67	0.008937	0.059253	0.384188	69	0.0080872	0.2169706	0.6413396	38	0.009935	0.039946	0.346489	70	0.006657	0.808144	1
Metabolism	Biosynthesis of other secondary metabolites	Butirosin and neomycin biosynthesis	313	0.0010564	9	0.0012	0.396143	1	15	0.0017581	0.0394694	0.1983337	2	0.000523	0.91288	1	11	0.001046	0.555431	1
Metabolism	Biosynthesis of other secondary metabolites	Flavonoid biosynthesis	1	3.375E-06	0	0	1	1	1	0.0001172	0.0287972	0.1523219	0	0	1	1	0	0	1	1
Metabolism	Biosynthesis of other secondary metabolites	Isoquinoline alkaloid biosynthesis	461	0.001556	19	0.002534	0.027735	0.222989	13	0.0015237	0.5689502	1	1	0.000261	0.997509	1	17	0.001617	0.47047	1
Metabolism	Biosynthesis of other secondary metabolites	Penicillin and cephalosporin biosynthesis	29	9.788E-05	2	0.000267	0.166398	0.69004	0	0	1	1	2	0.000523	0.053755	0.401096	5	0.000476	0.003276	0.041768
Metabolism	Biosynthesis of other secondary metabolites	Phenylpropanoid biosynthesis	446	0.0015053	10	0.001334	0.692845	1	29	0.003399	5.333E-05	0.0021439	7	0.00183	0.354592	0.997506	33	0.003138	7.55E-05	0.001711
Metabolism	Biosynthesis of other secondary metabolites	Tropane, piperidine and pyridine alkaloid biosynthesis	722	0.0024369	27	0.003601	0.030948	0.23925	32	0.0037506	0.0121109	0.1014288	9	0.002353	0.587132	1	32	0.003043	0.120264	0.489555
Metabolism	Carbohydrate metabolism	Galactose metabolism	5782	0.0195154	152	0.020275	0.326527	0.921027	221	0.0259025	2.025E-05	0.0010174	64	0.016732	0.907613	1	225	0.021398	0.084422	0.391412
Metabolism	Carbohydrate metabolism	Glyoxylate and dicarboxylate metabolism	5470	0.0184623	146	0.019474	0.266261	0.836224	179	0.0209798	0.0455306	0.2178964	79	0.020654	0.169785	0.676505	188	0.017879	0.684653	1
Metabolism	Carbohydrate metabolism	Starch and sucrose metabolism	10716	0.0361686	245	0.03268	0.95425	1	342	0.0400844	0.0278139	0.1523219	130	0.033987	0.778068	1	335	0.031859	0.993366	1
Metabolism	Energy metabolism	Sulfur metabolism	2908	0.0098151	103	0.013739	0.000554	0.010425	104	0.0121894	0.0160837	0.1293127	55	0.014379	0.004032	0.071111	94	0.00894	0.835771	1
Metabolism	Global and overview maps	Fatty acid metabolism	5286	0.0178413	125	0.016673	0.792067	1	185	0.0216831	0.0045556	0.0601264	85	0.022222	0.02576	0.309507	159	0.015121	0.98708	1
Metabolism	Glycan biosynthesis and metabolism	Glycosaminoglycan degradation	548	0.0018496	11	0.001467	0.81895	1	29	0.003399	0.0015129	0.0276449	4	0.001046	0.923551	1	41	0.003899	8.63E-06	0.00044
Metabolism	Glycan biosynthesis and metabolism	Glycosphingolipid biosynthesis - ganglio series	439	0.0014817	9	0.0012	0.781125	1	20	0.0023441	0.031527	0.1624853	1	0.000261	0.996683	1	26	0.002473	0.008466	0.071964
Metabolism	Glycan biosynthesis and metabolism	Glycosphingolipid biosynthesis - globo series	566	0.0019104	19	0.002534	0.132954	0.628663	29	0.003399	0.0024193	0.0347346	4	0.001046	0.934297	1	31	0.002948	0.012635	0.095462
Metabolism	Glycan biosynthesis and metabolism	Lipopolysaccharide biosynthesis	2385	0.0080498	77	0.010271	0.020102	0.181243	59	0.0069151	0.8968591	1	22	0.005752	0.960542	1	102	0.0097	0.033286	0.197914
Metabolism	Glycan biosynthesis and metabolism	Other glycan degradation	2481	0.0083739	42	0.005602	0.998047	1	86	0.0100797	0.0481689	0.2202793	22	0.005752	0.975462	1	129	0.012268	1.66E-05	0.000676
Metabolism	Glycan biosynthesis and metabolism	Peptidoglycan biosynthesis	9014	0.030424	273	0.036415	0.001594	0.021362	283	0.0331692	0.0727138	0.2815525	135	0.035294	0.045345	0.366541	370	0.035188	0.002474	0.036051
Metabolism	Lipid metabolism	Fatty acid biosynthesis	5206	0.0175713	124	0.01654	0.766287	1	179	0.0209798	0.009773	0.093007	84	0.021961	0.024734	0.309507	146	0.013885	0.998912	1
Metabolism	Lipid metabolism	Fatty acid degradation	2817	0.0095079	61	0.008137	0.905595	1	95	0.0111346	0.0675926	0.2717221	48	0.012549	0.035184	0.341701	67	0.006372	0.999854	1
Metabolism	Lipid metabolism	Linoleic acid metabolism	148	0.0004995	3	0.0004	0.725873	1	9	0.0010549	0.0281405	0.1523219	1	0.000261	0.853923	1	5	0.000476	0.606465	1
Metabolism	Lipid metabolism	Sphingolipid metabolism	1379	0.0046544	36	0.004802	0.447962	1	46	0.0053915	0.1740779	0.5467134	20	0.005229	0.33079	0.997466	65	0.006182	0.014297	0.103835
Metabolism	Lipid metabolism	Steroid biosynthesis	4	1.35E-05	0	0	1	1	0	0	1	1	2	0.000523	0.000983	0.023829	0	0	1	1
Metabolism	Lipid metabolism	Steroid hormone biosynthesis	38	0.0001283	2	0.000267	0.249894	0.79728	2	0.0002344	0.2993665	0.7428724	0	0	1	1	4	0.00038	0.044918	0.234954
Metabolism	Metabolism of cofactors and vitamins	Biotin metabolism	2461	0.0083064	76	0.010137	0.047396	0.344633	74	0.0086732	0.3687797	0.79704	39	0.010196	0.11588	0.556513	89	0.008464	0.443091	1
Metabolism	Metabolism of cofactors and vitamins	Folate biosynthesis	3026	0.0102133	114	0.015206	2.74E-05	0.000786	101	0.0118378	0.0745946	0.2815525	59	0.015425	0.001554	0.033507	129	0.012268	0.020735	0.136451
Metabolism	Metabolism of cofactors and vitamins	Nicotinate and nicotinamide metabolism	3481	0.0117491	97	0.012939	0.179608	0.69004	126	0.0147679	0.0062814	0.074268	51	0.013333	0.198453	0.716841	126	0.011983	0.423108	1
Metabolism	Metabolism of cofactors and vitamins	Porphyrin and chlorophyll metabolism	5613	0.018945	151	0.020141	0.231794	0.763779	188	0.0220347	0.0203706	0.1410703	86	0.022484	0.06274	0.41971	215	0.020447	0.133158	0.512533
Metabolism	Metabolism of cofactors and vitamins	Thiamine metabolism	2916	0.0098421	101	0.013472	0.001255	0.018011	113	0.0132443	0.0011869	0.0265085	49	0.01281	0.040995	0.346489	109	0.010366	0.303152	0.882626
Metabolism	Metabolism of cofactors and vitamins	Ubiquinone and other terpenoid-quinone biosynthesis	1605	0.0054172	56	0.00747	0.011488	0.128284	60	0.0070323	0.0269552	0.1523219	35	0.00915	0.002375	0.046067	69	0.006562	0.062473	0.310839
Metabolism	Metabolism of other amino acids	Cyanoamino acid metabolism	1958	0.0066086	48	0.006403	0.608141	1	79	0.0092593	0.0021521	0.033274	38	0.009935	0.010113	0.150915	85	0.008084	0.035976	0.197914
Metabolism	Metabolism of other amino acids	D-Alanine metabolism	1361	0.0045936	25	0.003335	0.962649	1	42	0.0049226	0.3458954	0.7811795	25	0.006536	0.053558	0.401096	79	0.007513	2.07E-05	0.000704
Metabolism	Metabolism of other amino acids	D-Arginine and D-ornithine metabolism	15	5.063E-05	1	0.000133	0.319176	0.921027	0	0	1	1	0	0	1	1	3	0.000285	0.014761	0.103835
Metabolism	Metabolism of terpenoids and polyketides	Biosynthesis of ansamycins	1013	0.0034191	27	0.003601	0.419058	1	38	0.0044538	0.0629028	0.2580297	13	0.003399	0.546407	1	53	0.00504	0.003857	0.045833
Metabolism	Metabolism of terpenoids and polyketides	Carotenoid biosynthesis	61	0.0002059	5	0.000667	0.019115	0.181243	1	0.0001172	0.8317998	1	1	0.000261	0.547391	1	2	0.00019	0.642047	1
Metabolism	Metabolism of terpenoids and polyketides	Geraniol degradation	189	0.0006379	2	0.000267	0.953529	1	10	0.0011721	0.0482203	0.2202793	0	0	1	1	4	0.00038	0.905801	1
Metabolism	Metabolism of terpenoids and polyketides	Terpenoid backbone biosynthesis	4550	0.0153571	120	0.016006	0.334502	0.921027	166	0.0194562	0.0014593	0.0276449	66	0.017255	0.184308	0.701093	205	0.019496	0.000399	0.007398
Metabolism	Xenobiotics biodegradation and metabolism	Benzoate degradation	255	0.0008607	7	0.000934	0.466923	1	14	0.0016409	0.0168833	0.1305209	5	0.001307	0.235069	0.814345	14	0.001331	0.072648	0.344655
Metabolism	Xenobiotics biodegradation and metabolism	Chlorocyclohexane and chlorobenzene degradation	19	6.413E-05	0	0	1	1	4	0.0004688	0.0018828	0.0315366	0	0	1	1	3	0.000285	0.0283	0.174944
Metabolism	Xenobiotics biodegradation and metabolism	Ethylbenzene degradation	21	7.088E-05	1	0.000133	0.416224	1	0	0	1	1	0	0	1	1	3	0.000285	0.036866	0.197914
Metabolism	Xenobiotics biodegradation and metabolism	Nitrotoluene degradation	230	0.0007763	3	0.0004	0.932001	1	8	0.0009376	0.3445068	0.7811795	1	0.000261	0.949704	1	14	0.001331	0.036371	0.197914
Metabolism	Xenobiotics biodegradation and metabolism	Toluene degradation	6	2.025E-05	0	0	1	1	3	0.0003516	0.0004472	0.0149824	0	0	1	1	2	0.00019	0.017174	0.116783
Organismal Systems	Digestive system	Protein digestion and absorption	245	0.0008269	3	0.0004	0.948499	1	7	0.0008204	0.560844	1	1	0.000261	0.958618	1	18	0.001712	0.003148	0.041768
Organismal Systems	Endocrine system	Estrogen signaling pathway	120	0.000405	1	0.000133	0.953864	1	8	0.0009376	0.0231608	0.1410703	1	0.000261	0.789784	1	6	0.000571	0.25424	0.774103
Organismal Systems	Endocrine system	Progesterone-mediated oocyte maturation	120	0.000405	1	0.000133	0.953864	1	8	0.0009376	0.0231608	0.1410703	1	0.000261	0.789784	1	6	0.000571	0.25424	0.774103
Organismal Systems	Excretory system	Vasopressin-regulated water reabsorption	1	3.375E-06	0	0	1	1	0	0	1	1	1	0.000261	0.01291	0.178898	0	0	1	1
Organismal Systems	Immune system	Antigen processing and presentation	120	0.000405	1	0.000133	0.953864	1	8	0.0009376	0.0231608	0.1410703	1	0.000261	0.789784	1	6	0.000571	0.25424	0.774103
Organismal Systems	Immune system	NOD-like receptor signaling pathway	126	0.0004253	1	0.000133	0.960443	1	9	0.0010549	0.0109766	0.0959259	1	0.000261	0.805557	1	9	0.000856	0.036074	0.197914

*TH: healthy tongue coating sample;
TD: tongue coating sample with halitosis;
SH: healthy saliva sample;
SD: saliva sample with halitosis.

Table S8-1 Functional gene abundances in hydrogen sulfide metabolic process in tongue coating samples

KO number	Gene name	Gene description	Gene_TH*_abundance	Gene_TD*_abundance	Ratio(Gene_TD_abundance/ Gene_TH_abundance)	↑ / ↓ /− (cutoff=2)
K00955	cysNC	bifunctional enzyme CysN/CysC [EC:2.7.7.4 2.7.1.25];	1.03282E-05	1.3838E-05	1.3	−
K00956	cysN	sulfate adenylyltransferase subunit 1 [EC:2.7.7.4];	0.000217222	0.000215965	1.0	−
K00957	cysD	sulfate adenylyltransferase subunit 2 [EC:2.7.7.4];	0.000132342	0.000117335	0.9	−
K00860	cysC	adenylylsulfate kinase [EC:2.7.1.25];	9.38931E-08	2.06995E-06	22.0	↑
K00394	aprA	adenylylsulfate reductase, subunit A [EC:1.8.99.2];	4.13129E-06	7.66647E-07	0.2	↓
K00390	cysH	phosphoadenosine phosphosulfate reductase [EC:1.8.4.8];	0.000124596	0.00011538	0.9	−
K00380	cysJ	sulfite reductase (NADPH) flavoprotein alpha-component [EC:1.8.1.2];	0.000261868	0.000295504	1.1	−
K00381	cysI	sulfite reductase (NADPH) hemoprotein beta-component [EC:1.8.1.2];	0.000262478	0.000251077	1.0	−
K00392	sir	sulfite reductase (ferredoxin) [EC:1.8.7.1]	5.63358E-07	5.36653E-07	1.0	−
K00640	cysE	serine O-acetyltransferase [EC:2.3.1.30];	0.000388107	0.000351585	0.9	−
K01738	cysK	cysteine synthase A [EC:2.5.1.47];	0.00037177	0.000296578	0.8	−
K12339	cysM	cysteine synthase B [EC:2.5.1.47];	1.87786E-07	7.66647E-08	0.4	↓
K01739	metB	cystathionine gamma-synthase [EC:2.5.1.48];	0.00030196	0.000241877	0.8	−
K17217	mccB	cystathionine gamma-lyase / homocysteine desulfhydrase [EC:4.4.1.1 4.4.1.2]	0	0	NaN	−
K01697	cbs	cystathionine beta-synthase [EC:4.2.1.22];	0	7.66647E-08	NaN	↑
K01760	metC	cystathionine beta-lyase [EC:4.4.1.8];	0.000292571	0.000223823	0.8	−
K14155	patB	cystathione beta-lyase [EC:4.4.1.8]	0.000289989	0.000197718	0.7	−
K00812	aspB	aspartate aminotransferase [EC:2.6.1.1];	0.000104691	7.01866E-05	0.7	−
K00813	aspC	aspartate aminotransferase [EC:2.6.1.1];	0.000189617	0.000174451	0.9	−
K11358	yhdR	aspartate aminotransferase [EC:2.6.1.1];	2.74168E-05	5.45853E-05	2.0	↑
K01011	sseA	thiosulfate/3-mercaptopyruvate sulfurtransferase [EC:2.8.1.1 2.8.1.2];	2.52103E-05	1.65979E-05	0.7	−
K02439	glpE	thiosulfate sulfurtransferase [EC:2.8.1.1]	2.53511E-06	1.53329E-06	0.6	−

*TH: healthy tongue coating sample;
TD: tongue coating sample with halitosis.

Table S8-2 Functional gene abundances in hydrogen sulfide metabolic process in saliva samples

KO number	Gene name	Gene description	Gene_SH*_abundance	Gene_SD*_abundance	Ratio(gene_SD_abundance/ gene_SH_abundance)	↑/↓/− (cutoff=2)
K00955	cysNC	bifunctional enzyme CysN/CysC [EC:2.7.7.4 2.7.1.25];	5.97456E-06	8.06678E-06	1.4	−
K00956	cysN	sulfate adenylyltransferase subunit 1 [EC:2.7.7.4];	0.000212223	0.000151112	0.7	−
K00957	cysD	sulfate adenylyltransferase subunit 2 [EC:2.7.7.4];	0.000142043	9.42456E-05	0.7	−
K00860	cysC	adenylylsulfate kinase [EC:2.7.1.25];	5.04892E-07	7.98691E-07	1.6	−
K00394	aprA	adenylylsulfate reductase, subunit A [EC:1.8.99.2];	6.73189E-07	1.43764E-06	2.1	↑
K00390	cysH	phosphoadenosine phosphosulfate reductase [EC:1.8.4.8];	0.000118229	6.99654E-05	0.6	−
K00380	cysJ	sulfite reductase (NADPH) flavoprotein alpha-component [EC:1.8.1.2];	0.000284591	0.000201829	0.7	−
K00381	cysI	sulfite reductase (NADPH) hemoprotein beta-component [EC:1.8.1.2];	0.000241086	0.000200312	0.8	−
K00392	sir	sulfite reductase (ferredoxin) [EC:1.8.7.1]	1.77554E-05	8.30639E-06	0.5	↓
K00640	cysE	serine O-acetyltransferase [EC:2.3.1.30];	0.000374714	0.000343917	0.9	−
K01738	cysK	cysteine synthase A [EC:2.5.1.47];	0.00029553	0.000337607	1.1	−
K12339	cysM	cysteine synthase B [EC:2.5.1.47];	6.73189E-07	3.19477E-07	0.5	↓
K01739	metB	cystathionine gamma-synthase [EC:2.5.1.48];	0.000295025	0.000289446	1.0	−
K17217	mccB	cystathionine gamma-lyase / homocysteine desulfhydrase [EC:4.4.1.1 4.4.1.2]	5.04892E-07	3.19477E-07	0.6	−
K01697	cbs	cystathionine beta-synthase [EC:4.2.1.22];	0	0	NaN	−
K01760	metC	cystathionine beta-lyase [EC:4.4.1.8];	0.000318755	0.000279143	0.9	−
K14155	patB	cystathione beta-lyase [EC:4.4.1.8]	0.000141538	0.000193842	1.4	−
K00812	aspB	aspartate aminotransferase [EC:2.6.1.1];	4.1906E-05	8.60191E-05	2.1	↑
K00813	aspC	aspartate aminotransferase [EC:2.6.1.1];	0.000192616	0.000153349	0.8	−
K11358	yhdR	aspartate aminotransferase [EC:2.6.1.1];	2.52446E-05	4.4567E-05	1.8	−
K01011	sseA	thiosulfate/3-mercaptopyruvate sulfurtransferase [EC:2.8.1.1 2.8.1.2];	7.82583E-06	2.63568E-05	3.4	↑
K02439	glpE	thiosulfate sulfurtransferase [EC:2.8.1.1]	8.41487E-07	2.87529E-06	3.4	↑

*SH: healthy saliva sample;
SD: saliva sample with halitosis.

Table S9 Bacterial candidates contributed to hydrogen sulphide-related functional variations

Gene name	Gene description	Taxonomic origin
cysM	cysteine synthase B	Acinetobacter calcoaceticus
		Burkholderia pseudomallei
		Cronobacter sakazakii
		Enterobacter aerogenes
		Escherichia coli
		Pseudomonas putida
		Pseudomonas syringae
		Shigella flexneri
		Yersinia enterocolitica
cbs	cystathionine beta-synthase	Lactobacillus plantarum
apsB	aspartate aminotransferase	Enterococcus faecalis
		Enterococcus faecium
		Peptoclostridium difficile
		Pseudomonas syringae
		Streptococcus mutans
		Streptococcus pneumoniae
		Streptococcus sanguinis
yhdR	aspartate aminotransferase	Bacillus subtilis
sseA	thiosulfate/3-mercaptopyruvate sulfurtransferase	Clostridium botulinum
		Cronobacter sakazakii
		Enterobacter aerogenes
		Escherichia coli
		Mesorhizobium loti
		Methylobacterium radiotolerans
		Morganella morganii
		Mycobacterium leprae
		Ochrobactrum anthropi
		Peptoclostridium difficile
		Proteus mirabilis
glpE	thiosulfate sulfurtransferase	Acinetobacter calcoaceticus
		Cronobacter sakazakii
		Enterobacter aerogenes
		Escherichia coli
		Haemophilus influenzae
		Morganella morganii
		Proteus mirabilis
		Pseudomonas putida
		Pseudomonas syringae
		Shigella flexneri
		Vibrio parahaemolyticus
		Yersinia enterocolitica
cysC	adenylylsulfate kinase	Escherichia coli
		Lactobacillus plantarum
		Pseudomonas syringae
		Shigella flexneri
		Yersinia enterocolitica

Oral species (Human Oral Microbiome Database)

Other human microbiota (not oral)
